# Towards Property Profiling: SYNTHESIS and SAR Probing of New Tetracyclic Diazaphenothiazine Analogues

**DOI:** 10.3390/ijms222312826

**Published:** 2021-11-26

**Authors:** Anna Empel, Andrzej Bak, Violetta Kozik, Malgorzata Latocha, Alois Cizek, Josef Jampilek, Kinga Suwinska, Aleksander Sochanik, Andrzej Zieba

**Affiliations:** 1 Department of Organic Chemistry, Faculty of Pharmaceutical Sciences in Sosnowiec, Medical University of Silesia in Katowice, Jagiellońska 4, 41-200 Sosnowiec, Poland; anna.empel00@gmail.com; 2 Institute of Chemistry, University of Silesia, Szkolna 9, 40-007 Katowice, Poland; violetta.kozik@us.edu.pl; 3 Department of Cell Biology, Faculty of Pharmaceutical Sciences in Sosnowiec, Medical University of Silesia in Katowice, Jedności 9, 41-200 Sosnowiec, Poland; mlatocha@sum.edu.pl; 4 Department of Infectious Diseases and Microbiology, Faculty of Veterinary Medicine, University of Veterinary Sciences Brno, Palackeho 1946/1, 61242 Brno, Czech Republic; cizeka@vfu.cz; 5 Department of Analytical Chemistry, Faculty of Natural Sciences, Comenius University, Ilkovicova 6, 84215 Bratislava, Slovakia; josef.jampilek@gmail.com; 6 Department of Chemical Biology, Faculty of Science, Palacky University Olomouc, Slechtitelu 27, 78371 Olomouc, Czech Republic; 7 Faculty of Mathematics and Natural Sciences, Cardinal Stefan Wyszyński University, K. Woycickiego 1/3, 01-938 Warszawa, Poland; k.suwinska@uksw.edu.pl; 8 Center for Translational Research and Molecular Biology of Cancer, Maria Skłodowska-Curie National Research Institute of Oncology, Wybrzeże AK 15, 44-101 Gliwice, Poland; aleksander.sochanik@io.gliwice.pl

**Keywords:** phenothiazine, azaphenothiazines, antiproliferative activity, antibacterial activity, lipophilicity, pharmacophore mapping, similarity-activity landscape index

## Abstract

A series of new tertiary phenothiazine derivatives containing a quinoline and a pyridine fragment was synthesized by the reaction of 1-methyl-3-benzoylthio-4-butylthioquinolinium chloride with 3-aminopyridine derivatives bearing various substituents on the pyridine ring. The direction and mechanism of the cyclization reaction of intermediates with the structure of 1-methyl-4-(3-pyridyl)aminoquinolinium-3-thiolate was related to the substituents in the 2- and 4-pyridine position. The structures of the compounds were analyzed using ^1^H, ^13^C NMR (COSY, HSQC, HMBC) and X-ray analysis, respectively. Moreover, the antiproliferative activity against tumor cells (A549, T47D, SNB-19) and a normal cell line (NHDF) was tested. The antibacterial screening of all the compounds was conducted against the reference and quality control strain *Staphylococcus aureus* ATCC 29213, three clinical isolates of methicillin-resistant *S. aureus* (MRSA). In silico computation of the intermolecular similarity was performed using principal component analysis (PCA) and hierarchical clustering analysis (HCA) on the pool of structure/property-related descriptors calculated for the novel tetracyclic diazaphenothiazine derivatives. The distance-oriented property evaluation was correlated with the experimental anticancer activities and empirical lipophilicity as well. The quantitative shape-based comparison was conducted using the CoMSA method in order to indicate the potentially valid steric, electronic and lipophilic properties. Finally, the numerical sampling of similarity-related activity landscape (SALI) provided a subtle picture of the SAR trends.

## 1. Introduction

The meaningful pathway of rational drug discovery is the search for new properties and applications of known (marketed) drugs and/or structural modifications of their basic fragments [1]. The introduction of new substituents, pharmacophore groups, and the creation of hybrid systems with other biologically active structures often leads to a change in the strength and direction of the pharmacological (biological) interactions.

Phenothiazines are an important class of tricyclic heterocycles widely used in medicinal chemistry—aminoalkyl phenothiazines are known neuroleptic and antihistamines [2]. Several different structural modifications of the phenothiazine system were carried out, mostly by introducing various types of substituents to the thiazine nitrogen atom, and the replacement of benzene rings with nitrogen heterocycles as well [3,4]. The synthesized new phenothiazine and azaphenothiazine derivatives showed diverse biological properties including antimicrobial, antitumor, antiviral, antituberculosis, antimalarial and anti-inflammatory ones [5,6,7,8,9,10]. Earlier works also described the synthesis of a number of tetracyclic quinobenzothiazine derivatives that revealed their antitumor as well as antibacterial properties [11,12,13,14,15]. Structural modifications of the basic fragment of quinobenzothiazines made it possible to generate derivatives with antiproliferative and antibacterial activities comparable to the marketed drugs.

In recent years, the medicinal chemist’s intuition (or serendipity) at the ‘presynthesis’ stage could be effectively aided by the computer-assisted molecular design (CAMD) approaches, that became almost ‘part and parcel’ of the exhaustive transformation of the compound topology/topography into the property-based chemical space. Strictly speaking, the chemical composition can be structurally coded by calculated high-level descriptors or represented by measured property data [16]. The quantitative potency modeling and ADMET-tailored property prediction or production strategies are expected to nominate the potent candidates in the hit → lead → seed → drug discovery process [17]. Accordingly, SAR-related mining of the descriptor-based feature/structural space seems crucial in order to minimize the likelihood of late attrition in drugs; however, the simple transition from complex biological relationships to straightforward quantitative-SAR models is regarded as a ‘triumph of hope over experience’ [18]. A paradox seen in chemistry is that new compounds are usually synthesized in the hope of finding novel properties using a ‘trial and error’ approach. In fact, the property profiling, not the synthesis itself, should be a merit of contemporary chemistry, as was noted by Hammond [19]. Thus, in silico mapping of molecular descriptors to targeted functionalities can support the synthetic efforts at the decision-making phases of the rational drug design [20].

On the whole, the computer-aided estimation of the drug-host recognition phenomena can be theoretically dichotomized into on-target (receptor-dependent) and off-target (receptor-independent) approaches, respectively [21]. Practically, the ligand-related procedures produce the hypothetical pharmacophore that is based on the straightforward concept of substituent interchangeability and complementarity in the congeneric set of molecules, where similar size, shape and charge distributions should impose comparable influence on binding affinity and pharmacological profile as well [22,23]. Unarguably, the molecular similarity is still the core of many SAR-based procedures assuming that small changes in molecular structure induce small variations in activity and vice versa. The similarity-driven procedures that engage fuzzy logic algorithms (e.g., comparative molecular shape analysis) have been proposed to roughly estimate the complex physiological reality [24]. Despite some drawbacks, the distance-related similarity examination of structurally alike compounds still contribute considerably to the quantitative or qualitative SAR specification [25,26]. In particular, the numerical approximation of the smooth (homogenous) areas and/or the sharp (heterogeneous) activity cliffs in the form of structure-activity landscape indexes (SALI) can provide hints that can be implemented at the synthetic phase [27]. The smart management of SALI-driven data can help to modulate pharmacological response as well as to optimize ADMET-friendly drug properties (minimization of unwanted side effects) [28].

The principal objective of the presented study was the design, synthesis and in vitro characterization of the anticancer profile for a novel series of tetracyclic diazaphenothiazine derivatives with the functionalized pyridine subunit. Following common practice, the experimental data were investigated in silico; therefore, the intermolecular similarity-based examination was conducted using the principal component analysis (PCA) and hierarchical clustering analysis (HCA) on the pool of structure/property-related descriptors calculated for novel tetracyclic diazaphenothiazine derivatives. The distance-oriented property evaluation for the congeneric series of compounds was correlated with anticancer experimental activities and empirical lipophilicity profile, respectively. Moreover, the newly constructed adducts were subjected to the quantitative shape comparison (CoMSA) with the generation of an averaged pharmacophore pattern to illustrate the key 3D steric/electronic/lipophilic features. Eventually, the quantitative sampling of the similarity-related activity landscape (SALI) provided a subtle picture of favorable and disallowed structural modifications that are valid for determining the activity cliffs. Obviously, we take a critical view of the activity spectrum for a new set of tetracyclic diazaphenothiazine derivatives with the functionalized pyridine subunit; however, the investigated series might be subsequently structurally modified in order to optimize the drug’s anticancer activity and selectivity.

## 2. Results and Discussion

### 2.1. Chemistry—Design and Synthesis

In an earlier work we described the synthesis of 5-methyl-12*H*-quino[3,4-*b*]pyrido[5,6-*e*][1,4]thiazinium chloride **3a** and its derivative containing an ethyl group at the quinoline nitrogen atom. These compounds were obtained by reactions of 5,12-(dialkyl) thioquinanthrenediinium *bis*-chloride with 3-aminopyridine [15]. X-ray analysis of derivative **3a** showed that this compound has a completely flat tetracyclic pyridoquinothiazinium system. It seemed that this could have a significant (large, decisive) effect on the biological properties of this type of compound. The phenothiazine and azaphenothiazine derivatives reported in the literature had a bent thiazine system. Obviously, the changes in the conformation of molecules can have significant effects on receptor fit, drug distribution in the body, and metabolism as well. It has been the basis of research into synthesis of new derivatives and the assessment of their biological properties.

**Scheme 1:**
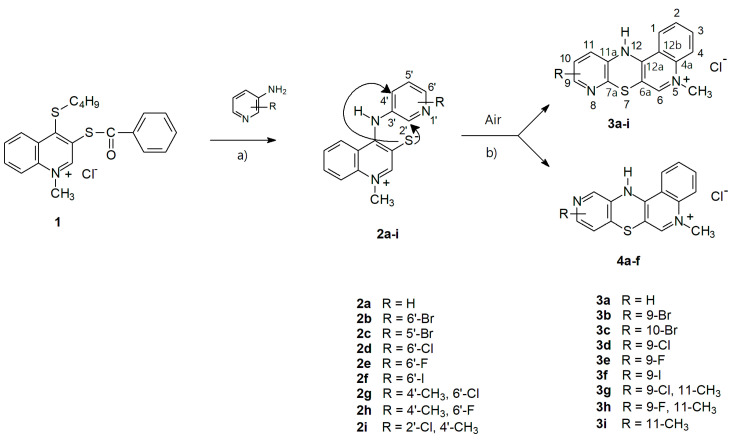
Reactions of salt **1** with 3-aminopyridines containing hydrogen atoms in the 2- and 4-position of the pyridine ring. Reagents and conditions: (**a**) pyridine, 80 °C (**b**) pyridine, HCl, air, 80 °C.

In this paper we report the synthesis of a series of novel tetracyclic pyridoquinothiazinium derivative **3** by reacting 1-methyl-3-benozoylthio-4-(butylthio)quinolinium chloride **1** with 3-aminopyridines bearing various types of substituents (Br, Cl, F, I, CH_3_, OCH_3_) at different positions on the pyridine ring. The mechanism and reaction direction depend on the presence of halogen atoms in the 2- and 4-positions of the 3-aminopyridine. The reaction proceeds by a nucleophilic attack of the exocyclic nitrogen of the aminopyridine on the carbon at the 4-quinoline position and on the acyl carbon. This leads to the substitution of the thiobutyl group with a pyridylamino group, cleavage of the thioacyl bond leading to the formation of the thiolate function in the 3-quinolinone position and the production of 1-methyl-4-(pyridylamino)quinolinio-3-thiolates **2**. The quinolinio-3-thiolates **2** containing additional halogen atoms in the pyridine ring showed very high reactivity and underwent further transformations in the solution, which made it impossible to isolate them and determine their structure by NMR. At the second stage of the reaction, the quinoline-3-thiolates **2** were cyclized to product **3**. Earlier studies indicated nucleophilic character of the reaction at this stage [11,12]. In the case of quinoline-3-thiolane **2** having a pyridylamino group in the 4-quinoline position, the nucleophilic attack of the thiolate sulfur atom could occur at the 2- or 4-pyridyl position. Depending on the cyclization direction, such a reaction should lead to two different types of derivatives, **3** or **4**, containing a nitrogen atom in the 8- or 10-position of the pyridoquinothiazinium tetracyclic system (see Scheme 1).

The expected pyridoquinothiazinium chloride **3** was generated with the highest yield when the reactions were carried out in pyridine solution at the temperature of 80 °C. A further increase in temperature did not augment the yield, while the presence of additional products was observed in the postreaction mixtures. In the case of quinolinio-3-thiolates **2a**–**f**, when the hydrogen atom was present in the 2- and 4-position of the pyridyl moiety, cyclization was preferred via substitution of the hydrogen atom in the 2-position of the pyridine ring, leading to products **3a**–**f**, that contain an endocyclic nitrogen atom in the 8-position of the pyridoquinothiazinium system. Only traces of the 4-isomers were found in the reaction mixtures, resulting from the substitution of the hydrogen atom in the 4-position of the pyridine ring. So far, it has not been possible to isolate them in pure form from postreaction mixtures. Their presence has been confirmed on the basis of TLC chromatography of postreaction mixtures and MS and ^1^H NMR spectra of crude reaction products. In the case of thiolates **2g**–**i** bearing a methyl group in the 4-pyridyl position, the cyclization reaction only led to the corresponding pyridoquinothiazines **3g**–**i** by substituting a hydrogen or halogen atom in the 2-pyridyl position.

**Scheme 2:**
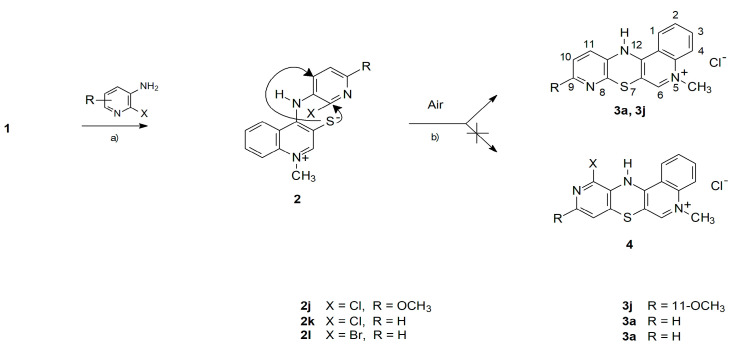
Reactions of salt **1** with 3-aminopyridines containing halogen atoms in the 2-position of the pyridine ring. Reagents and conditions: (**a**) pyridine, 80 °C (**b**) pyridine, HCl, air, 80 °C.

In the case of the reactions of salt **1** with 2-chloro- and 2-bromo-3-aminopyridine, the **2j**–**l** quinolinio-3-thiolates formed at the first stage of the reaction contained a halogen atom in the 2-pyridyl position, and a hydrogen atom in the 4-pyridyl position (see Scheme 2). With this arrangement of substituents on the pyridine ring, the cyclization reaction took place solely by substituting a halogen atom with a thiolate sulfur atom, leading to compound **3a** and **3j**, respectively. In the postreaction mixtures, no trace amounts of products **4** were found, resulting from the substitution of the hydrogen atom in the 4-pyridyl position. The course of the reaction did not depend on the presence of oxygen in the reaction medium.

**Scheme 3:**
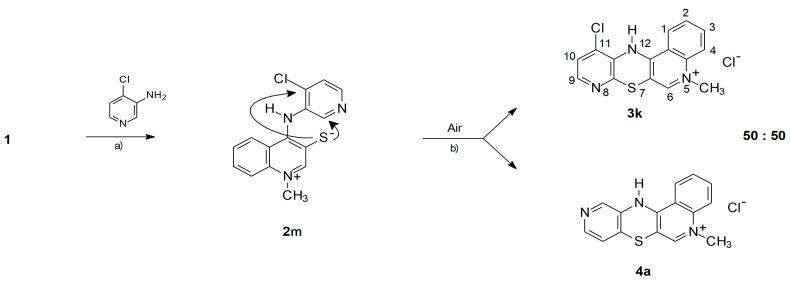
Reactions of salt **1** with 4-chloro-3-aminopyridine in the presence of atmospheric oxygen. Reagents and conditions: (**a**) pyridine, 80 °C (**b**) pyridine, HCl, air, 80 °C.

The reaction of salt **1** with 4-chloro-3-aminopyridine was different when the **2m** quinolinio-3-thiolate formed at the first stage of the reaction contained a halogen atom in the 4-position of the pyridine ring, and a hydrogen atom in the 2-position (see Scheme 3). It seemed interesting whether the direction of the reaction would be more influenced by the type of leaving group or the nature of the reaction center. It turned out that, when the reaction was carried out in the presence of atmospheric oxygen, the cyclization of quinoline-3-thiolane **2m** occurs by both the substitution of a hydrogen atom in the 2-pyridyl position and the substitution of a chlorine atom in the 4-pyridyl position. This resulted in the formation of two pyridoquinothiazinium salts, **3k** and **4a**, that contain an endocyclic nitrogen atom in the 8- or 10-position of the pyridoquinothiazinium system as shown in Scheme 3. ^1^H NMR spectra indicates a mixture composition close to equimolar.

The mixture of **3k** and **4a** isomers could not be separated by chromatographic methods. Isomer **4a** is a previously undescribed pyridoquinothiazinium derivative, therefore attempts have been made to obtain it using various mechanisms of the formation of **3m** and **4a** compounds:

- The cyclization to **3k** takes place as a nucleophilic substitution of a hydrogen atom. The hydride anion is a very difficult leaving group and its substitution in nucleophilic substitution reactions requires the presence of an oxidizing agent. In this case, it is atmospheric oxygen;
- The cyclization to **4a** takes place as a nucleophilic substitution of a halogen atom by a thiolate sulfur atom. It is a direct process and does not require the presence of an oxidant in the reaction medium.

**Scheme 4:**
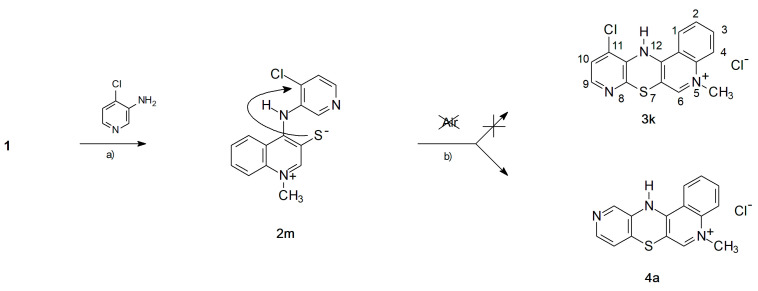
Reaction of salt **1** with 4-chloro-3-aminopyridine the under anaerobic conditions. Reagents and conditions: (**a**) pyridine, 80 °C (**b**) pyridine, HCl, 80 °C.

To block the reaction pathway to **3k** formation, the reaction of salt **1** with 4-chloro-3-aminopyridine was performed under anaerobic conditions. Atmospheric oxygen was removed from the reaction mixture by bubbling argon purge (see Scheme 4). This reaction was selective and gave derivative **4a** with a yield of 80%.

### 2.2. Spectroscopic Structural Analysis

The structures of compounds **3a**–**j** and **4a** were confirmed by ESI-HRMS spectrometry and ^1^H as well as ^13^C NMR spectroscopy. An important diagnostic element for confirming the reaction route and the compounds’ structure was the multiplicity and integration of proton signals from the pyridine ring. In the case of some derivatives, a significant broadening of the signals in the proton spectra was observed which is probably due to the dynamic phenomena that take place in the solution consisting of the exchange of the amine proton between the pyridine and thiazine nitrogen atom. For the selected derivatives, using two-dimensional techniques (COSY, HSQC, HMBC), a complete assignment of signals was made for all atoms in the proton and carbon spectra.

In the spectra generated for all the new compounds by ESI-HRMS spectrometry, the signal corresponding to the mass of pyridoquinothiazinium cations was produced with accuracy to three decimal places relative to the theoretically determined value, which undoubtedly confirms their elemental composition.

### 2.3. X-ray Structural Analysis

**Figure 1:**
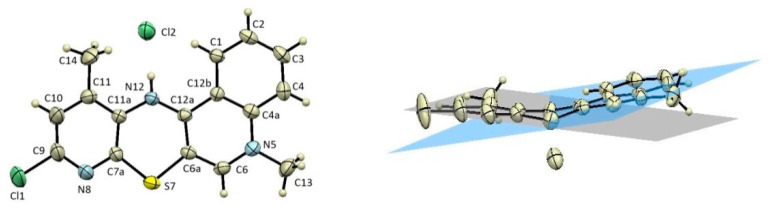
X-ray structure of 9-chloro-5,11-dimethyl-12*H*-quino[3,4-*b*]pyrido[5,6-*e*][1,4]thiazinium chloride molecule (**3g**).

X-ray analysis of the compounds not only confirmed the established NMR methods, but also showed their solid state arrangement. By crystallization from ethanol, it was possible to obtain single crystals of the derivatives **3g** and **3j**. The X-ray structure of the derivative **3g** is shown in Figure 1.

**Figure 2:**
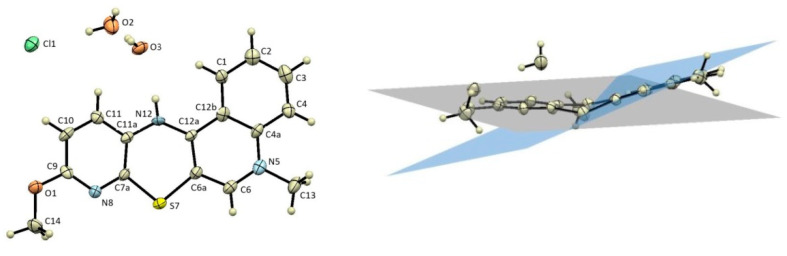
X-ray structure of 9-methoxy-5-methyl-12*H*-quino[3,4-*b*] pyrido[5,6-*e*][1,4]thiazinium chloride molecule (**3j**).

The molecule of the 9-chloro-5,11-dimethyl-12*H*-quino[3,4-*b*]pyrido[5,6-*e*][1,4]thiazinium chloride **3g** is bent along the axis defined by the nitrogen and sulfur atom of the ring of thiazine at an angle of 160.56°. The angle formed by the atoms in the C6a-S7-C7a thiazine ring has a value of 100.2°, while between the atoms C11a-N12-C12a α = 124.8°. The X-ray structure of the derivative **3j** is shown in Figure 2.

The molecule of the 9-methoxy-5-methyl-12*H*-quino[3,4-*b*]pyrido[5,6-*e*][1,4]thiazinium chloride **3j** is bent along the axis defined by the nitrogen and sulfur atom of the thiazine ring at an angle of 154.34°. The angle formed by the atoms C6a-S7-C7a in the thiazine ring is 100.2°, while between the atoms C11a-N12-C12a it is 125.1°. The values of the thiazine ring angles in the compound **3j** are almost identical to those in the compound **3g**. Most of the structural parameters of **3g** and **3j** molecules correspond well with the parameters produced for the previously described tetracyclic structure of 5-methyl-12*H*-quino[3,4-*b*][1,4]benzothiazinium chloride [11]. However, the previously described 5-ethyl-12*H*-pyrido[5,6-*e*]quino[3,4-*b*][1,4]thiazinium chloride with no additional substituents on the pyridine ring, had a completely flat tetracyclic structure [15]. The molecules of the newly synthesized isomers **3g** and **3j** are bent along the axis defined by the nitrogen and sulfur atoms of the thiazine ring. It seems that the presence of additional substituents has a significant impact on the geometry of the molecule, which may also influence the biological activity of the compounds obtained.

### 2.4. In Vitro Cytotoxic Activity

The previously described tetracyclic quinobenzothiazinium derivatives showed interesting anticancer properties [11,12,13,14]. Literature reports suggest that the mechanism of their antitumor activity is based on DNA intercalation [12]. The newly synthesized pyridoquinothiazinium salts are structural analogs of quinobenzothiazines in which the benzene ring has been replaced with a pyridine ring. The structure of the synthesized compounds was additionally modified by introducing various substituents into the pyridine ring. One of the goals of this research was to investigate how such a structural modification would affect the cytotoxic properties. For all the derivatives obtained, tests of cytotoxic activity against tumor cells (A549, T47D, SNB-19 lines) and the NHDF normal cell line were carried out. The values of the IC_50_ parameters are presented in Table 1. In fact, the compounds possess similar IC_50_ values against cancer cell lines vs. the normal cell line (NHDF). On the other hand, a similar phenomenon is commonly observed in chemotherapy as unwanted side effects. The main aim of the research was the design, synthesis and in vitro characterization of the anticancer profile for a novel series of tetracyclic diazaphenothiazine derivatives with the functionalized pyridine subunit that might be subsequently structurally modified in order to optimize the anticancer drug activity and selectivity.

Cytotoxic activity depended both on the nature of the substituents and their position in the molecule. The presence of additional substituents (-Br, -Cl, -F, -CH_3_, -OCH_3_) on the pyridine ring significantly increased the cytotoxic activity compared to the unsubstituted derivative **3a**. The derivative **4a**, containing the endocyclic nitrogen atom in the 10-position of the tetracyclic system, showed a higher cytotoxic activity than the derivative **3a**, in which the endocyclic nitrogen atom is in the 8-position. The compounds that showed the highest activity in relation to all tested cell lines were: **3d**, **3e** and **3j** derivatives, bearing a chlorine atom, a fluorine atom or a methoxy group in the 9-position of the pyridoquinothiazinium tetracyclic system, respectively. Compounds with 9- or 10-position bromine atoms showed lower activity. The compound with the lowest cytotoxic activity was the **3f** derivative, which has an iodine atom in the 9-position of the pyridoquinothiazinium system. It should be noted that many of the derivatives obtained showed greater cytotoxic activity for the tested tumor cell lines than cisplatinum (used as a reference molecule). All derivatives **3** and **4a** also show cytotoxic properties against the normal NHDF cell line.

The structural and empirical data of tetracyclic diazaphenothiazine derivatives **3a**–**j** and **4a** are reported in Table 1.

### 2.5. In Vitro Antimicrobial Activity

The biological screening of the analyzed compounds was performed against the reference and quality control strain *Staphylococcus aureus* ATCC 29213, three clinical isolates of methicillin-resistant *S. aureus* (MRSA) [29]. All data, expressed as minimum inhibitory concentrations (MICs), are summarized in Table 2. Based on these results, it can be stated that most of the compounds did not show any antistaphylococcal activity. Exceptions are compounds **3c** and **3e**, which have MICs in the range of 12.5–42.1 μM against MRSA isolates and thus show activity comparable to the used ampicillin standard. As MICs against MRSA isolates were, in fact, comparable with the MIC values observed against methicillin-susceptible *S. aureus* ATCC 29213, it could be assumed that the presence of *mecA* gene did not affect the activity of these compounds [30].

### 2.6. Similarity-Oriented Property Mapping

**Figure 3:**
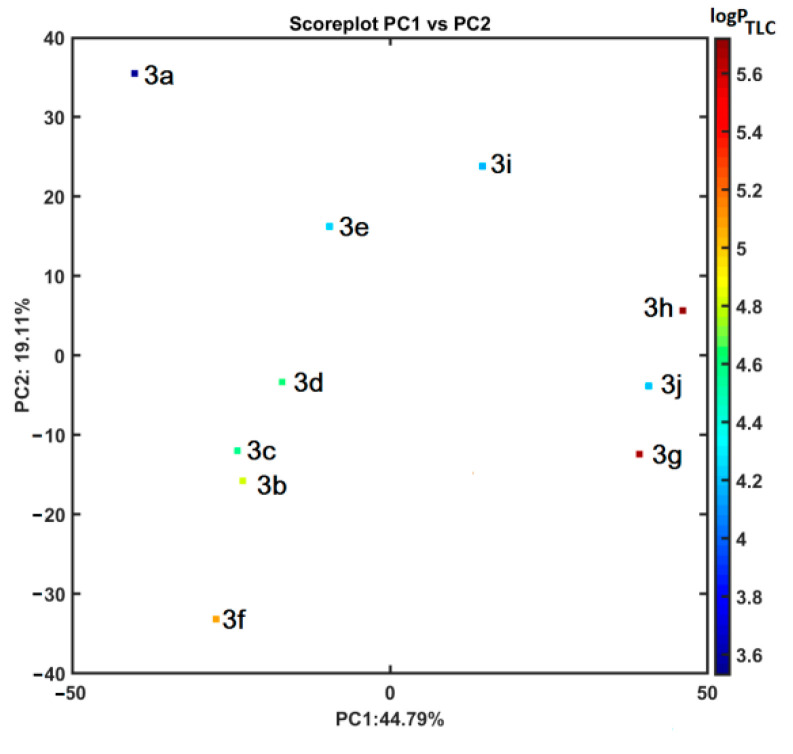
Projection of molecules **3a**–**j** on plane defined by PC1 and PC2 with corresponding experimental lipophilicity in logarithmic scale. Colors code for log*P* values.

The distance-guided property assessment was performed using principal component analysis (PCA) and hierarchical clustering analysis (HCA) on the ensemble of 2622 descriptors retrieved from Dragon 6.0 software. The derived data were organized into matrix X_10×2622_, where rows present molecules (objects) and columns represent numerical parameters (variables), respectively. The descriptor-based matrix was centered and standardized, because the original variables differed considerably. The resulting number of important principal components (PCs) was specified, taking into account the percentage of the modeled variance. The first three PCs accounted for 74.42% of the total data variance, whereas the first two PCs described 63.91%. The corresponding score plot with the projection of molecules **3a**–**j** on the plane PC1 vs. PC2 color-coded according to the experimental lipophilicity (logP_TLC_) is presented in Figure 3. Not surprisingly, the unsubstituted compound **3a** is located separately on the principal component plane. Interestingly, the analogues **3g**–**j** with a methylated and methoxylated pyridine subunit are separated from the remaining ones on the positive area of PC1. Moreover, the most potent antiproliferative molecules **3d** and **3e** with chlorine and fluorine substituents are grouped together in the range of −20 < PC1 < −10. Obviously, the lipophilicity of diazaphenothiazine derivatives is dependent on the lipophilic character of pyridine substituents. Roughly speaking, more lipophilic molecules with the experimental logP_TLC_ > 4.5 are located in the negative area of PC2 as shown in Figure 3.

**Figure 4:**
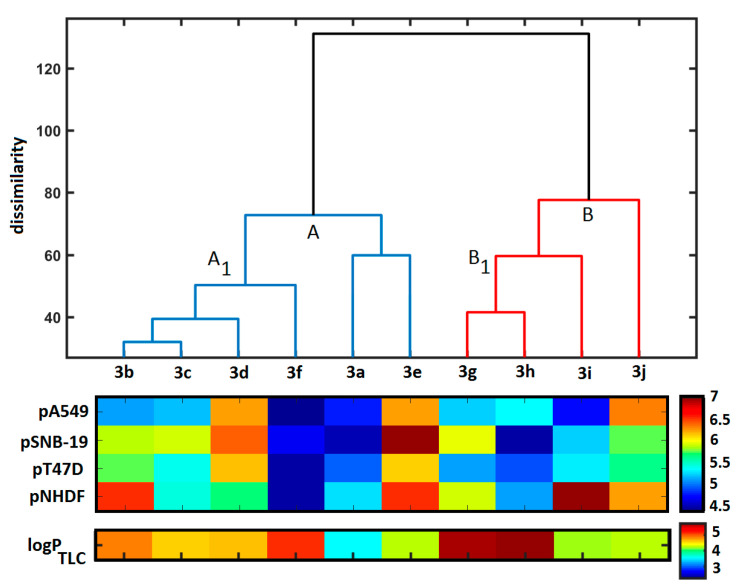
Dendrogram of compounds **3a**–**j** in descriptor-based space with color-coded map of experimental activities and experimental lipophilic values in logarithmic scale.

In order to investigate in depth, the (dis)similarities between objects (molecules) in the multidimensional descriptor-based space, the exploratory HCA analysis was conducted as well. The clustering tendency of HCA leads to a sub-optimal grouping of objects that is mostly related to the procedure engaged for cluster linkage [31]. Due to the hierarchical nature of the HCA method, the results are presented as dendrograms generated in the Euclidean-based distance with the Ward linkage algorithm, where the OX axis presents the sequence of objects/parameters and the OY axis specifies the dissimilarity [32]. Unfortunately, the interpretability of the extracted data structure is not simple in the multidimensional variable space; therefore, the dendrogram might be augmented with a color-coded map of the experimental activity and lipophilicity data in the logarithmic scale as presented in Figure 4.

Generally, two main clusters (A and B) can be distinguished on the dendrogram in Figure 4 that proved our previous PCA results (see Figure 3). On the whole, the molecules **3g**–**j** (red lines) varied noticeably from the remaining compounds (blue lines) of the dataset. A concurrent investigation of the dendrogram objects (sorted according to the Ward linkage method) and the color-coded map of the empirical data (activities and lipophilic profile) indicated that there is no evident structure-activity (SAR) or structure-property (SPR) relationship, respectively. Basically, subclusters A_1_ and B_1_ are characterized by relatively high values of logP_TLC_ parameter, when compared to the remaining objects.

**Figure 5:**
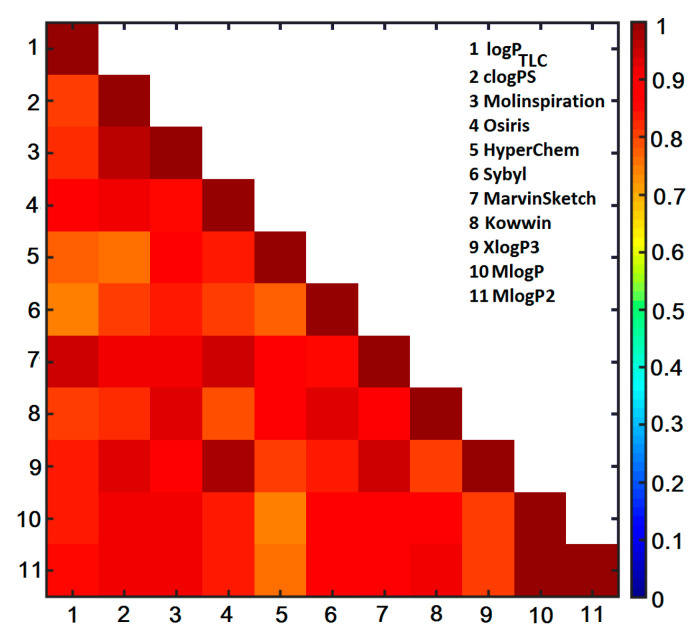
Matrix of linear correlation coefficients between empirical logP_TLC_ and clogP calculated by different programs.

In order to examine the lipophilic profile of the investigated compounds, the empirically determined logP_TLC_ parameters (see Table 1) were compared with the theoretically approximated lipophilicity coefficients (clogP) using an ensemble of in silico predictors, e.g., clogP, Molinspiration, Osiris, HyperChem 7.0, Sybyl-X, MarvinSketch 15, ACD/ChemSketch 2015, Dragon 6.0, Kowwin, XlogP3, ChemDraw and ACD/Percepta programs. Subsequently, the deduced clogP parameters were cross-compared with the experimental logP_TLC_ and inter-correlated with each other as presented in Figure 5. A good correlation was obtained (r = 0.94) for the MarvinSketch-made clogP values and the empirical logP_TLC_ was recorded, respectively. In fact, the clogP values averaged over the set of programs produced the value of r = 0.88, while the median resulted in r = 0.83 with the experimental data. A quite high inter-correlation was observed among the programs for lipophilicity specification. Obviously, some variations in clogP values resulted from different in silico algorithms (atom/fragment- or descriptor-based) implemented in the programs and/or training data employed in the training step [33].

Moreover, the recursive variable elimination PLS-based algorithm (IVE-PLS) as well as stepwise Matlab procedure applied on the integrated clogP matrix (X_10×10_) and experimental logP_TLC_ indicated MarvinSketch estimator to be a valid contributor to the linear QSPR model.

**Figure 6:**
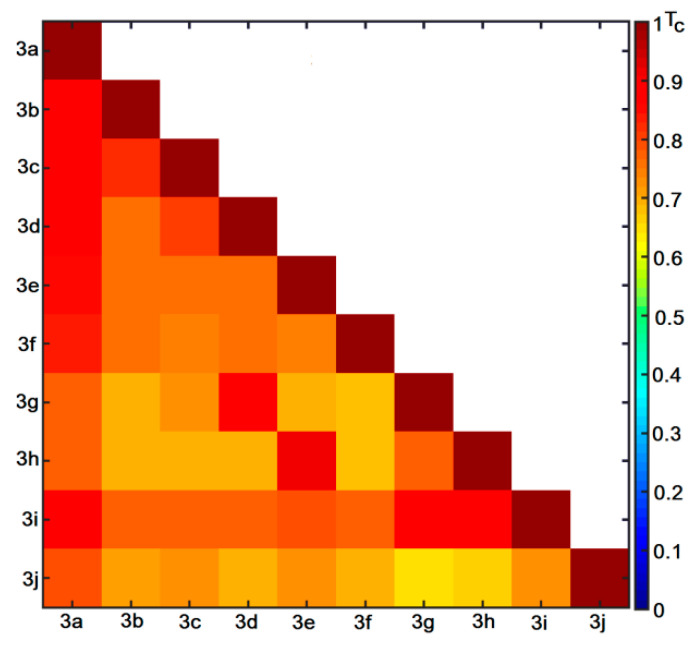
Triangular matrix of Tanimoto coefficients for compounds **3a**–**j**.

The examination of similarity-based SAR trends (the continuity areas and/or activity cliffs) using the planar image of the structure-activity landscape indexes (SALI) is dependent largely on the availability of the structurally-related molecules (chemotypes) with discernible activity variations [34]. Hence, the distribution of Tanimoto coefficients (T_C_) was analyzed for the triangular T_10×10_ matrix (see Figure 6) indicating that the majority of molecules have relatively high similarity values (T_C_ ≥ 0.70). On the whole, molecules **3g**, **3h** and **3j** are characterized by lower T_C_ values as compared to the remaining compounds, which confirms our previous PCA and HCA findings (see Figure 3 and Figure 4). On the other hand, the most potent antiproliferative molecules **3d** and **3e** are marked as fairly similar to the remaining compounds in the database.

**Figure 7:**
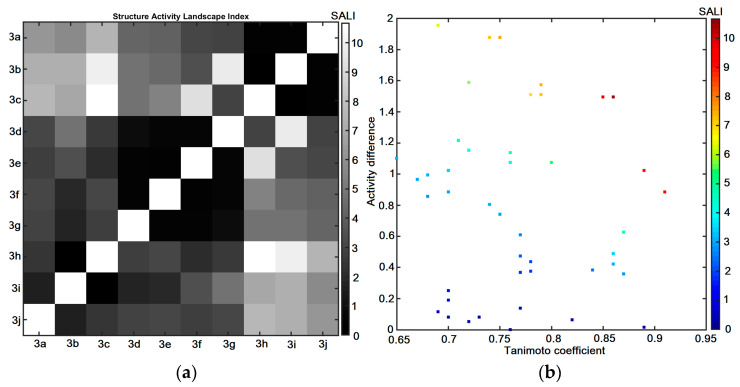
Grayscale SALI plane with molecules **3a**–**j** ordered according to increasing pA549 potential (**a**) and neighborhood plot (**b**).

The symmetrical grayscale heatmap of SALI numerical values for the structurally-related compounds **3a**–**j** is presented in Figure 7a, where the axes correspond to molecules sorted according to the increasing pA549 activity potential. Bright spots represent the high values of SALI parameters, whereas black spots specify the minimal values of SALI, where small structural modifications induce pretty small changes of molecular activity [35]. The structural relatedness between pairs of compounds in the function of the activity variations is illustrated by the color-coded neighborhood plot in Figure 7b. Based on the simultaneous interpretation of Figure 7a,b one can conclude that the most potent antiproliferative molecules **3d**, **3e** and **3j** are characterized by the highest SALI values with unsubstituted analogue **3a**. Interestingly, two additional pairs of pretty similar chlorine/fluorine-based molecules (**3d** vs. **3g** and **3e** vs. **3h**) are accompanied by dark-brown spots in Figure 7b, that can potentially form the activity cliff—the introduction of a methyl group in the pyridine subunit is manifested by the demolition of activity (magic methyl phenomenon) [36]. Consequently, further profound samplings of the indicated SAR-variations (T > 0.85 and ΔpA549 > 1) seem advisable.

### 2.7. Probability-Driven Pharmacophore Probing

**Figure 8:**
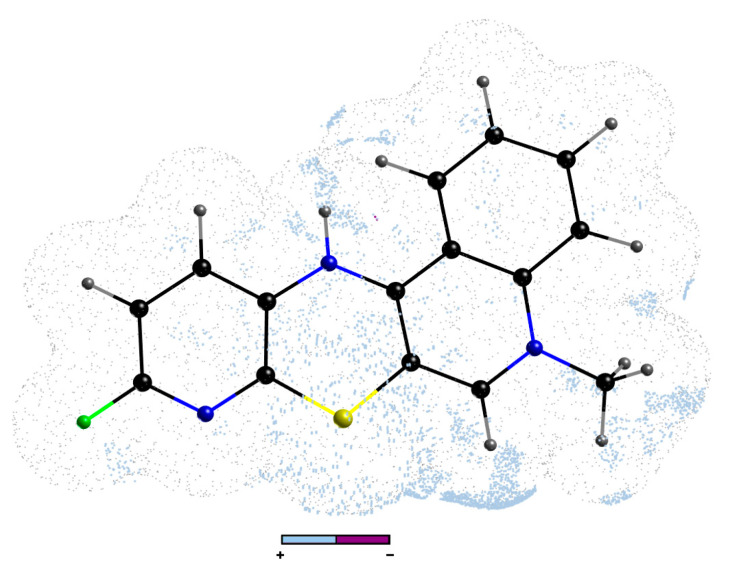
The 3D pattern specified by CoMSA IVE-PLS procedure for 7/3 training/test samplings. Colors code the positive (bright points) and negative (dark points) contributions on the molecular surface. The potent compound **3e** is plotted as a reference molecule.

The spatial distributions of the ligand electronic/steric/lipophilic properties that might be valid for the guest–host composition can be determined by the systematic probing of the functional group changes and the corresponding activity variations, respectively [37]. Unfortunately, the structural relatedness with the biological response can be specified basically for the homogeneous set of molecules that usually share the common substructure (chemotype) [38]. In other words, the introduction of the pharmacophore concept in QSAR studies for a non-congeneric series of structures is a rather elusive and enigmatic operation. Additionally, the prediction/validation of the modeled property/activity is firmly dependent on the molecule training/test separation. In fact, there are no universal principles for splitting the ensemble of molecules into training/test subsets so as to keep robust and predictive SAR models. Thus, the repetitive and interchangeable training/test subset division was proposed for the probability-driven pharmacophore probing based on the stochastic model validation (SMV) algorithm [39]. The systematically generated training/test subset population with the ratio 7:3 was examined using the comparative molecular surface analysis (CoMSA) for pA549 potency. The distribution of the molecular frequency in the test subset for the preferable statistic parameters ($q_{cv}^{2}$ > 0.5) indicted that the most potent antiproliferative compounds **3d** and **3e** were noticeably overrepresented in the training population, whereas molecules **3h** and **3g** were frequently distributed in the test set. Not surprisingly, the preferential elimination of the most active molecules from the test subset resulted in the production of the potentially robust SAR models. Eventually, the selection-driven pharmacophore pattern was produced using the robust models according to the IVE-PLS-based algorithm that was described elsewhere [40]. The graphical illustration of the descriptor-selected areas with the preselected cut-off value of 0.6 that potentially (un)favorably contribute to CoMSA models is presented in Figure 8.

Noticeably, a densely populated set of steric/electrostatic features was indicated on the averaged molecular surface for the set of the analyzed compounds; therefore, the direct mapping of the spatial pharmacophore-based areas into the corresponding pseudoreceptor model that potentially harbors putative ligands is rather a tricky task. Interestingly, the mostly favorable contributions of the 3D pattern (marked by the bright colors) that embrace mainly a methyl group of the quinolone’s skeleton, as well as the sulfur and nitrogen linkers are indicated, respectively. The positively charged areas of hydrogen atoms directly attached to nitrogen atoms were depicted as the beneficial contributors that can potentially form hydrogen bonds with the hypothetical amino acid residue in the receptor/enzyme pocket. It seems that α-halogenated pyridine ring contributes favorably to the observed activity profile according to the general tendency, where substituents can be ranked as follows: F > Cl > Br > I. Obviously, the steric and electronic features of the halogen-based substituents can be related to the increasing atomic F🡢I volume/surface (van der Waals radius) and electronegativity (Pauling scale), respectively. The generated pharmacophore pattern shed only some light on the recorded variations in the activities of tetracyclic diazaphenothiazine derivatives due to the limited number of compounds; therefore, new analogues need to be synthesized and analyzed.

## 3. Materials and Methods

### 3.1. Chemistry

Melting points are uncorrected. NMR spectra were recorded using a Bruker Ascend

600 spectrometer (Bruker, Billerica, MA, USA). To assign the structures, the following 2D experiments were employed: ^1^H-^13^C gradient selected HSQC and HMBC sequences. Standard experimental conditions and standard Bruker programs were used. The ^1^H NMR and ^13^C NMR spectral data are given relative to the TMS signal at 0.0 ppm. HR mass spectra were recorded with Bruker Impact II (Bruker, Billerica, MA, USA).

#### 3.1.1. Synthesis of 5-Methyl-12*H*-quino [3,4-*b*]pyrido[5,6-*e*][1,4]thiazinium Chloride **3a**–**j**

To a suspension of 1 mmol (0.448 g) of 1-methyl-3-benzoylthio-4-(butylthio) quinolinium chloride in 6 mL of anhydrous pyridine, 2.5 mmol of the corresponding 3-aminopyridine was added. The suspension was heated at T = 80 °C with constant stirring, allowing atmospheric oxygen to enter the reaction mixture. After the reactants had reacted completely, the mixture was cooled to room temperature and the resulting precipitate was filtered off under reduced pressure and washed 5 times (5 mL each) with ether. The crude product was purified by alumina chromatography column using chloroform: ethanol (10:1, *v*/*v*) as eluent.

*5-methyl-12H-quino[3,4-b]pyrido[5,6-e][1,4]thiazinium chloride* (**3a**): Yield: 80%; ^1^H NMR (CD_3_OD, 600 MHz) δ (ppm): 4.10 (s, 3H, NCH_3_), 7.00–6.99 (d.d, ^3^*J* = 4.8 Hz, ^3^*J* = 7.8 Hz, 1H, H10), 7.33–7.34 (d.d, ^3^*J* = 7.8 Hz, ^4^*J* = 1.2 Hz, 1H, H11), 7.74–7.75 (m, 1H, H2), 7.91–7.92 (d.d, ^3^*J* = 4.8 Hz, ^4^*J* = 1.2 Hz, 1H, H9), 7.96–7.98 (m, 2H, H3, H4), 8.36 (s, 1H, H6), 8.40–8.41 (m, 1H, H1); ^13^C NMR (CD_3_OD, 150.9 MHz) δ (ppm): 42.29 (NCH_3_), 108.46 (C4a), 115.86 (C12b), 118.33 (C4), 122.79 (C1), 123.14 (C10), 124.26 (C11), 128.35 (C2), 133.44 (C11a), 134.77 (C3), 139.24 (C12a), 141.41 (C7a), 143.04 (C6), 146.42 (C9), 150.96 (C6a); ESI-HRMS Calcd for C_15_H_12_N_3_S ([M]^+^): 266.0746, Found: 266.0749.
*9-bromo-5-methyl-12H-quino[3,4-b]pyrido[5,6-e][1,4]thiazinium chloride* (**3b**): Yield: 75%; ^1^H NMR (CD_3_OD, 600 MHz) δ (ppm): 4.22 (s, 3H, NCH_3_), 7.10–7.16 (d, ^3^*J* = 8.1 Hz 1H, H10), 7.37–7.42 (d, ^3^*J* = 8.1 Hz, 1H, H11), 7.80–7.90 (m, 1H, H_arom_), 8.06–8.13 (m, 2H, H_arom_), 8.42–8.50 (m, 2H, H_arom_); ^13^C NMR (CD_3_OD, 150.9 MHz) δ (ppm): 42.47 (NCH_3_), 108.64 (C), 116.01 (C), 118.40 (C), 118.63 (C), 122.79 (C), 126.12 (C), 128.55 (C), 134.31 (C), 134.91 (C), 139.19 (C), 140.44 (C), 143.31 (C), 146.70 (C), 148.17 (C); ESI-HRMS Calcd for C_15_H_11_Br[^79^]N_3_S ([M]^+^): 343.9852, Found: 343.9849; ESI-HRMS Calcd for C_15_H_11_Br[^81^]N_3_S ([M]^+^): 345.9831, Found: 345.9828.
*10-bromo-5-methyl-12H-quino[3,4-b]pyrido[5,6-e][1,4]thiazinium chloride* (**3c**): Yield: 72%; ^1^H NMR (CD_3_OD, 600 MHz) δ (ppm): 4.22 (s, 3H, NCH_3_), 7.25–7.32 (m, 2H, H_arom_), 7.83–7.92 (m, 1H, H_arom_), 8.05–8.14 (m, 2H, H_arom_), 8.42–8.50 (m, 2H, H_arom_); ^13^C NMR (CD_3_OD, 150.9 MHz) δ (ppm): 42.37 (NCH_3_), 108.01 (C), 115.91 (C), 118.37 (C), 122.75 (C), 126.39 (C), 126.93 (C), 128.48 (C), 133.08 (C), 134.93 (C), 136.36 (C), 139.31 (C), 142.06 (C), 143.13 (C), 150.86 (C); ESI-HRMS Calcd for C_15_H_11_Br[^79^]N_3_S ([M]^+^): 343.9852, Found: 343.9849; ESI-HRMS Calcd for C_15_H_11_Br[^81^]N_3_S ([M]^+^): 345.9831, Found: 345.9828.
*9-chloro-5-methyl-12H-quino[3,4-b]pyrido[5,6-e][1,4]thiazinium chloride* (**3d**): Yield: 85%; ^1^H NMR (CD_3_OD, 600 MHz) δ (ppm): 4.23 (s, 3H, NCH_3_), 7.15–7.18 (d, *J* = 8.4Hz, 1H, H10), 7.38–7.41 (d, *J* = 8.4 Hz, 1H, H11), 7.85–7.90 (m, 1H, H2), 8.06–8.14 (m, 2H, H3, H4), 8.44–8.47 (m, 1H, H1), 8.48 (s, 1H, H6); ^13^C NMR (CD_3_OD, 150.9 MHz) δ (ppm): 42.35 (NCH_3_), 107.90 (C4a), 115.89 (C12b), 118.36 (C4), 122.76 (C1), 123.04 (C10), 126.72 (C11), 128.46 (C2), 132.67 (C11a), 134.93 (C3), 139.31 (C12a), 141.66 (C), 143.12 (C6), 146.86 (C), 150.91 (C6a); ESI-HRMS Calcd for C_15_H_11_ClN_3_S ([M]^+^): 300.0357, Found: 300.0352.
*9-fluoro-5-methyl-12H-quino[3,4-b]pyrido[5,6-e][1,4]thiazinium chloride* (**3e**): Yield: 69%; ^1^H NMR (CD_3_OD, 600 MHz) δ (ppm): 4.21 (s, 3H, NCH_3_), 6.70–6.82 (m, 1H, H_arom_), 7.54–7.60 (m, 1H, H_arom_), 7.84–7.89 (m, 1H, H_arom_), 8.05–8.12 (m, 2H, H_arom_), 8.44 (s, 1H, H6), 8.48–8.50 (m, 1H, H_arom_); ^13^C NMR (CD_3_OD, 150.9 MHz) δ (ppm): 42.25 (NCH_3_), 107.41 (C, *J*_C–F_ = 36.2 Hz), 107.78 (C), 115.77 (C), 118.31 (C), 122.79 (C), 128.34 (C), 129.53 (C, *J*_C–F_ = 7.5 Hz), 131.28 (C, *J*_C–F_ = 4.5 Hz), 134.90 (C), 139.09 (C, *J*_C–F_ = 15.1 Hz), 139.35 (C), 142.99 (C), 151.29 (C), 160.85 (C, *J*_C–F_ = 241.4 Hz); ESI-HRMS Calcd for C_15_H_11_FN_3_S ([M]^+^): 284.0652, Found: 284.0649.
*9-iodo-5-methyl-12H-quino[3,4-b]pyrido[5,6-e][1,4]thiazinium chloride* (**3f**): Yield: 78%; ^1^H NMR (CD_3_OD, 600 MHz) δ (ppm): 4.22 (s, 3H, NCH_3_), 7.04–7.09 (d, ^3^*J* = 8.4 Hz, 1H, H11), 7.49–7.53 (d, ^3^*J* = 8.4 Hz, 1H, H10), 7.83–7.90 (m, 1H, H_arom_), 8.05–8.13 (m, 2H, H_arom_), 8.44–8.46 (m, 1H, H1), 8.48 (s, 1H, H6); ^13^C NMR (DMSO_d-6_, 150.9 MHz) δ (ppm): 43.34 (NCH_3_), 107.24 (C), 111.99 (C) 116.24 (C), 119.35 (C), 124.45 (C), 125.98 (C), 126.85 (C), 128.64 (C), 134.27 (C), 135.30 (C), 139.23 (C), 142.55 (C), 143.95 (C), 150.33 (C); ESI-HRMS Calcd for C_15_H_11_IN_3_S ([M]^+^): 391.9713, Found: 391.9713.
*9-chloro-5,11-dimethyl-12H-quino[3,4-b]pyrido[5,6-e][1,4]thiazinium chloride* (**3g**): Yield: 61%; ^1^H NMR (CD_3_OD, 600 MHz) δ (ppm): 2.45 (s, 3H, 11CH_3_), 4.28 (s, 3H, NCH_3_), 7.14 (s, 1H, H10), 7.85–7.95 (m, 1H, H_arom_), 8.10–8.21 (m, 2H, H_arom_), 8.40–8.45 (m, 1H, H_arom_), 8.55–8.66 (m, 1H, H_arom_); ^13^C NMR (CD_3_OD, 150.9 MHz) δ (ppm): 15.53 (CH_3_), 42.60 (NCH_3_), 110.05 (C), 116.73 (C), 118.36 (C), 123.05 (C), 124.81 (C), 128.52 (C), 131.48 (C), 134.97 (C), 138.73 (C), 139.26 (C), 142.55 (C), 143.65 (C), 146.69 (C); 151.94 (C); ESI-HRMS Calcd for C_16_H_13_ClN_3_S ([M]^+^): 314.0513, Found: 314.0513.
*9-fluoro-5,11-dimethyl-12H-quino[3,4-b]pyrido[5,6-e][1,4]thiazinium chloride* (**3h**): Yield: 63%; ^1^H NMR (CD_3_OD, 600 MHz) δ (ppm): 2.50 (s, 3H, CH_3_), 4.28 (s, 3H, NCH_3_), 6.78 (s, 1H, H10), 7.88–7.95 (m, 1H, H_arom_), 8.11–8.20 (m, 2H, H_arom_), 8.40–8.45 (m, 1H, H_arom_), 8.61 (s, 1H, H6); ^13^C NMR (CD_3_OD, 150.9 MHz) δ (ppm): 16.015 (CH_3_, *J*_C–F_ = 1.5 Hz), 42.57 (C), 109.28 (C, *J*_C–F_ = 37.7 Hz), 116.63 (C), 118.35 (C), 123.08 (C), 128.46 (C), 130.08 (C), 134.97 (C), 139.27 (C), 140.08 (C, *J*_C–F_ = 16.6 Hz), 141.73 (C, *J*_C–F_ = 7.5 Hz), 143. 56 (C), 152.35 (C), 160.74 (C, *J*_C–F_ = 239.9 Hz); ESI-HRMS Calcd for C_16_H_13_FN_3_S ([M]^+^): 298.0809, Found: 298.0814.
*5,11-dimethyl-12H-quino[3,4-b]pyrido[5,6-e][1,4]thiazinium chloride* (**3i**): Yield: 65%; ^1^H NMR (CD_3_OD, 600 MHz) δ (ppm): 2.57 (s, 3H, 11-CH_3_), 4.33 (s, 3H, NCH_3_), 7.27–7.31 (d,^3^*J* = 5.4 Hz, 1H, H_pirydyl_), 7.91–7.00 (m, 1H, H_arom_), 7.08–7.13 (d, ^3^*J* = 5.4 Hz, 1H, H_pirydyl_), 7.14–7.20 (m, 1H, H_arom_), 7.2–7.25 (m, 1H, H_arom_), 7.47–7.52 (m, 1H, H_arom_), 8.74 (s, 1H, H6); ^13^C NMR (CD_3_OD, 150.9 MHz) δ (ppm): 16.02 (CH_3_), 42.86 (NCH_3_), 109.44 (C), 116.89 (C), 118.51 (C), 123.14 (C), 126.21 (C), 128.75 (C), 133.61 (C), 135.16 (C), 138.48 (C), 139.30 (C), 141.47 (C), 143.50 (C), 144.11 (C), 152.16 (C); ESI-HRMS Calcd for C_16_H_14_N_3_S ([M]^+^): 280.0903, Found: 280.0907.
*9-methoxy-5-methyl-12H-quino[3,4-b]pyrido[5,6-e][1,4]thiazinium chloride* (**3j**): Yield: 86%; ^1^H NMR (CD_3_OD, 600 MHz) δ (ppm): 3.83 (s, 3H, OCH_3_), 4.15 (s, 3H, NCH_3_), 6.40-6.52 (d, ^3^*J* = 8.4 Hz, 1H, H10), 7.38–7.42 (d, ^3^*J* = 8.4 Hz, 1H, H11), 7.80–7.83 (m, 1H, H2), 8.00–8.05 (m, 2H, H3, H4), 8.32 (s, 1H, H6), 8.43–8.46 (m, 1H, H1); ^13^C NMR (DMSO_d-6_, 150.9 MHz) δ (ppm): 42.90 (NCH_3_), 54.40 (OCH_3_), 106.25 (C4a), 109.22 (C10), 115.86 (C12b), 119.06 (C4), 124.86 (C1), 127.75 (C2), 128.14 (C11), 129.77 (C3), 135.08 (C), 137.25 (C12a), 139.26 (C11a), 143.0 (C6), 150.74 (C6a), 161.77 (C); ESI-HRMS Calcd for C_16_H_14_N_3_OS ([M]^+^): 296.0852, Found: 296.0861.

#### 3.1.2. Synthesis of 5-Methyl-12*H*-quino[3,4-*b*]pyrido[3,4-*e*][1,4]thiazinium Chloride **4a**

Argon was bubbled through a suspension (0.448 g) of 1-methyl-3-benzoylthio-4-(butylthio)quinolinium chloride and 2.5 mmol of 3-amino-4-chloropyridine in 6 mL of anhydrous pyridine for 30 min. The flask was sealed and the reaction was carried out at room temperature for 30 days while stirring the suspension on a magnetic stirrer. The resulting precipitate was then filtered off with suction and washed 5 times (5 mL each) with ether. The crude product was purified by alumina chromatography column using chloroform: ethanol (10:1, *v*/*v*) as eluent.

*5-methyl-5H-qujino[3,4-b]pyrido[3,4-e][1,4]thiazinium chloride* (**4a**): Yield: 80%; ^1^H NMR (CD_3_OD, 600 MHz) δ (ppm): 4.11 (s, 3H, NCH_3_), 6.96–6.97 (d, ^3^*J* = 4.8 Hz, 1H, H8), 7.75–7.77 (m, 1H, H2), 7.99–8.00 (m, 3H, H3, H4, H9), 8.15 (s, 1H, H11), 8.36 (s, 1H, H6), 8.43–8.45 (m, 1H, H1); ^13^C NMR (CD_3_OD, 150.9 MHz) δ (ppm): 42.34 (NCH_3_), 105.91 (C6a), 116.15 (C12b), 118.36 (C4), 121.25 (C8), 122.77 (C1), 128.39 (C2), 129.17 (C7a), 133.50 (C11a), 134.92 (C3), 137.12 (C11), 139.33 (C4a), 143.08 (C6), 146.99 (C9), 152.25 (C12a); ESI-HRMS Calcd for C_15_H_12_N_3_OS ([M]^+^): 266.0751, Found: 266.0749.
^1^H NMR and ^13^C NMR spectrum data are reported in Supplementary Materials (Figures S1–S22).

### 3.2. X-ray Structural Analysis

Crystal data for C_16_H_13_ClN_3_S+PCl^−^ (**3g**): M = 350.25, red needle, 0.048 × 0.056 × 0.359 mm, monoclinic, space group *P*2_1_/*n*, *a* = 5.1502(1), *b* = 23.5062(5), c = 12.6880(3), Å, *β* = 90.234(1)°, *V* = 1536.02(6), Å^3^, *Z* = 4, *D*_c_ = 1.515 g Pcm^−3^, *F*000 = 720, Bruker AXS D8 VENTURE DUO, Cu*Ka* radiation, *λ* = 1.54178 Å, *T* = 166(2), K, *θ*_max_ = 72.48°, 49,709 reflections collected, 3025 unique (*R*_int_ = 0.076). Final *GooF* = 1.03, *R* = 0.038, *wR* = 0.093, *R* indices based on 2447 reflections with *I* > 2*σ* (*I*) (refinement on *F*^2^), 205 parameters, 0 restraints. Lp and absorption corrections applied, *μ* = 3.365 mm^−1^. CCDC deposition number 2095596.

Crystal data for C_16_H_14_N_3_OS+PCl^−^P2H_2_O (**3j**): M = 367.84, red needle, 0.031 × 0.039 × 0.372 mm, monoclinic, space group *P*21/*n*, *a* = 11.548(1), *b* = 6.9221(8), *c* = 21.054(2), Å, *β* = 102.187(7)°, *V* = 1645.0(3), Å^3^, Z = 4, *Dc* = 1.534 gPcm^−3^, *F*000 = 786, Bruker AXS D8 VENTURE DUO, Cu*Ka* radiation, *λ* = 1.54178 Å, *T* = 150(2), K, *θ*_max_ = 62.47°, 13,217 reflections collected, 2453 unique (*R*_int_ = 0.097). Final *GooF* = 1.01, *R* = 0.075, *wR* = 0.161, R indices based on 1375 reflections with *I* > 2*σ*(*I*) (refinement on *F*^2^), 233 parameters, 0 restraints. Lp and absorption corrections applied, *μ* = 3.426 mm^−1^. CCDC deposition number 2095597.

### 3.3. Biological Evaluation

#### 3.3.1. Cell Culture

Compounds were evaluated for their antiproliferative activity using three cultured cell lines: A549 (human lung carcinoma, ATCC, Manassas, VA, USA), SNB-19 (human glioblastoma, DSMZ, German Collection of Microorganisms and Cell Cultures), T-47D (human breast cancer, ATCC, Manassas, VA, USA) and NHDF (normal human dermal fibroblasts, ATCC, Manassas, VA, USA). The cultured cells were kept at 37 °C and 5% CO_2_. The cells were seeded (1 × 10^4^ cells/well/100 μL DMEM supplemented with 10% FCS and streptomycin and penicillin) using 96-well plates (Corning). Cells were counted in a hemocytometer (Burker chamber) using a phase contrast Olympus IX50 microscope equipped with Sony SSC-DC58 AP camera and Olympus DP10 digital camera.

#### 3.3.2. Proliferation Assay

The antiproliferative effect of the compounds exerted on cancer and normal cells was determined using the Cell Proliferation Reagent WST-1 assay (Roche Molecular Biochemicals Mannheim, Germany). This assay is based on a colorimetric method using the enzymatic ability of viable cells to cause the bright red-colored stable tetrazolium monosodium salt [2-(4-iodophenyl)-3-(4-nitrophenyl)-5-(2,4-disulfophenyl)–2H-tetra-zolium] to transform into the dark red-colored soluble formazan. A greater number of viable cells resulted in a greater overall activity of mitochondrial dehydrogenases in the measured sample. An increase in the amount of formazan dye formed correlated with the number of metabolically active cells in the culture. The formazan dye produced by metabolically active cells was quantified by absorbance reading at appropriate wavelengths. The examined cells were exposed to the tested compounds (1 mg/mL DMSO stock) for 72 h at various concentrations (0.1 μg/mL–100 μg/mL). The control was performed in order to eliminate the DMSO effect at the concentration used. Cell cultures were incubated with WST-1 (10 μL) for 1 h. The absorbance of the samples was measured against a background control at 450 nm using a microplate reader with a reference wavelength at 600 nm. The obtained results are expressed as the means of at least two independent experiments performed in triplicate. The antiproliferative activity of the tested compounds were compared to cisplatinum. The values of IC_50_ (a concentration of a compound that is required for 50% inhibition) were calculated from the dose-response relationship with respect to control.

#### 3.3.3. In Vitro Antistaphylococcal Evaluation

The synthesized compounds were evaluated for in vitro antibacterial activity against representatives of multidrug-resistant bacteria and clinical isolates of methicillin-resistant *Staphylococcus aureus* (MRSA) 63718, SA 630, and SA 3202 that were obtained from the National Institute of Public Health (Prague, Czech Republic) [29,41]. *S. aureus* ATCC 29213 was used as a reference and quality control strain. Ampicillin (Sigma, St. Louis, MO, USA) was employed as the standard. Prior to testing, each strain was passaged onto nutrient agar (Oxoid, Basingstoke, UK) with 5% of bovine blood, and bacterial inocula were prepared by suspending a small portion of a bacterial colony in sterile phosphate-buffered saline (pH 7.2–7.3). The cell density was adjusted to 0.5 McFarland units using a densitometer (Densi-La-Meter, LIAP, Riga, Latvia). This inoculum was diluted to reach the final concentration of bacterial cells 5 × 10^5^ CFU/mL in the wells. The compounds were dissolved in DMSO (Sigma), and the final concentration of DMSO in the cation-adjusted Mueller–Hinton (CaMH) broth (Oxoid) did not exceed 2.5% of the total solution composition. The final concentrations of the evaluated compounds ranged from 256 to 0.008 μg/mL. The broth dilution micro-method, modified according to the NCCLS (National Committee for Clinical Laboratory Standards) guidelines in Mueller–Hinton (MH) broth, was used to determine the minimum inhibitory concentration (MIC) [42]. Drug-free controls, sterility controls, and controls consisting of MH broth and DMSO alone were included. The determination of results was performed visually after 24 h of static incubation in the darkness at 37 °C in an aerobic atmosphere. Each experiment was repeated at least three times. The results are reported in Table 2.

### 3.4. Molecular Modeling

#### 3.4.1. Model Building

CACTVS/csed and CORINA molecular generators were engaged to produce 3D structural geometries. The file format transformation was performed using OpenBabel (inter)change file format converter. Molecular modeling simulations were conducted using Sybyl-X 2.0 package (Certara) installed on a DELL workstation with Ubuntu 20.10 operating. MAXMIN2 module implemented in Sybyl-X was applied in order to optimize the initial compound spatial geometry with the standard Tripos force field (POWELL conjugate gradient algorithm) with a 0.01 kcal/mol energy gradient convergence criterion. The electrostatic potential values were calculated using Gasteiger–Hückel method. One 10-ordered atom probe superimposition was generated on the most active compound **3e** according to the active analogue approach (AAA) with FIT method to encompass the entire bonding topology (quinoline, pyridine and linkers) in the maximal common structure (MCS).

In order to simulate 20 × 20 to 40 × 40 self-organizing maps (SOMs) with a winning distance in the range from 0.2 to 2.0 in CoMSA analysis SONNIA software was employed. Cartesian (x, y, z) coordinates of the molecular surfaces and the corresponding potential values distributed on the molecular surface were used as input to SOM network to produce a 2D map of the electrostatic potential (MEP). Subsequently, the obtained maps were reshaped into a 400- to 1600-element vector and subjected to the PLS method implemented in our MATLAB software.

#### 3.4.2. Principal Component and Hierarchical Clustering Analysis

The human-friendly 2D/3D images of the molecular distribution in the experimental (FCS) and virtual (VCS) molecular space could be produced using the principal component analysis (PCA). Briefly speaking, PCA is the projection method that can transform model multidimensional data (mD) into 2D/3D space (scores and loadings) with a relatively small number of so-called principal components (PCs) produced to maximize the description of variance within the input data. The PCA model with *f* principal components for a data matrix *X* can be calculated as follows:

*X* = *TP^T^* + *E* (1)

where *X* is a data matrix with *m* objects and *n* variables, *T* is the score matrix with dimensions (*m* × *f*), *P^T^* is a transposed matrix of loadings with dimensions (*f* × *n*) and *E* is a matrix of the residual variance (*m* × *n*) that is not explained by the first *f* principal components. Generally, the first few principal components usually sufficiently describe data variance.

In order to examine the (dis)similarities between objects in the descriptor-based space the hierarchical clustering analysis (HCA) was combined with a color-coded map of empirical dataset [31,32]. In fact, the similarity measure (e.g., Euclidean distance) as well as the manner of resulting subcluster linkage (e.g., Ward’s algorithm) should be specified *a priori.* A dendrogram augmented with visual display of experimental data allows the direct interpretation of the produced clusters in terms of the original parameters, where OX axis presents the indices of the clustered objects while OY corresponds to the linkage distances between two objects linked, respectively.

#### 3.4.3. Theoretical Lipophilicity Evaluation

A variety of freely/commercially available in silico estimators can be applied to estimate the theoretical partition coefficients (clogP) using the additive type-dependent, atom-based or cumulative fragment-related lipophilicity contributions with correction factors calibrated on experimental training set, for instance, AlogPS, milogP, ClogP, HyperChem logP, MarvinSketch logP, Dragon MlogP, Kowwin, XlogP3, OSIRIS clogP. The redundant descriptor-based parameters of QSAR/QSPR studies were selected/eradicated using the modified version of the uninformative variable elimination (UVE–PLS) method, namely, iterative variable elimination (IVE–PLS) [39]. In short, the whole algorithm is composed of several recurrent stages: (i) standard PLS analysis with LOO-CV to evaluate the performance of the PLS model; (ii) elimination of the matrix column with the lowest *abs (mean (b)/std (b))* value; (iii) standard PLS analysis of the new matrix without the column eliminated in (ii); (iv) iterative repetition of (i)–(iii) to maximize $q_{cv}^{2}$ value.

#### 3.4.4. Similarity-Related Activity Landscape Index

The quantitative probing of the similarity-based structure-activity landscape index (SALI) can be numerically expressed according to the following equation:

(2) $$SALI_{x,y}=\frac{\left|A_{x}-A_{y}\right|}{1-sim\left(x,y\right)}$$

where $A_{x}$ and $A_{y}$ are the activity profiles for the *x*-th and *y*-th molecule and *sim*(*x*,*y*) is the pair-wise similarity metric. The fingerprint-based Tanimoto coefficient was chosen to estimate the pairwise molecular similarities as follows:

(3) $$T\left(x,y\right)=\frac{n_{xy}}{\left(n_{x}+n_{y}-n_{xy}\right)}$$

where $n_{xy}$ is the number of bits set into 1 shared in the fingerprint of the molecule *x* and *y*, $n_{x}$ is the number of bits set into 1 in the molecule *x*, $n_{y}$ is the number of bits set into 1 in the molecule *y*, respectively.

#### 3.4.5. Ligand-Based SAR Probing

The iterative IVE-PLS method was engaged to prune the original set of CoMSA descriptors as a filter to eliminate nonsignificant variables (probably noise data) and to specify the structural descriptors having the highest individual weightings for the biological activity [40]. The relative importance or weight of each informative descriptor is determined by the magnitude of the regression coefficient (*b*) giving a clear 3D picture of the regions that should be modified to modulate the biological activity. On the whole, the extraction of a column from the data matrix which is assigned with the lowest value of *abs (mean (b)/std (b))* slightly improves the $q_{cv}^{2}$ performance. The backward column elimination is recurrently repeated as long as the optimal number of variables included within the model is achieved—the moment of $q_{cv}^{2}$ deterioration indicates the number of the relevant columns, i.e., crucial variables to be incorporated into the final PLS model. Unlike in the standard procedure that displays such plots for a single training/test subset, an attempt was made to identify a common ensemble of variables which survived backward elimination and contributed importantly to activity simultaneously in all chosen models. The cumulative sum of common columns for chosen models was specified and normalized to the [0÷1] range. Subsequently, the group of columns with the value above the pre-chosen cutoff of 0.6 was selected. The relative contribution of each variable was weighted by the magnitude and the sign of the corresponding regression coefficient. The bright spheres delineated the spatial pattern where an atom or substituent was predicted to be positioned in order to increase the compound’s activity, while the dark polyhedra denoted the areas detrimental for the potency, probably due to steric hindrance or electrostatic factors.

## 4. Conclusions

We present here a new synthetic route leading to tetracyclic pyridoquinothiazine derivatives based on the reaction of 1-methyl-3-benzoylthio-4-(butylthio)quinolinium chloride with 3-aminopyridine derivatives bearing various substituents on the pyridine ring. The direction and mechanism of the cyclization of intermediates with the structure of 1-methyl-4-(3-pyridylamino)quinolinium-3-thiolate was dependent on the substituents in the 2- and 4-pyridine position. The structures of the compounds were confirmed by ^1^H, ^13^C NMR methods using advanced two-dimensional techniques (COSY, HSQC, HMBC). The direction of the cyclization reaction (formation of the thiazine ring) and the products structure was confirmed by X-ray diffraction analysis. Moreover, the biological activity of the obtained derivatives was investigated. The antiproliferative activity against tumor cell lines (A549, T47D and SNB-19) as well as a normal cell line (NHDF) was tested. The antibacterial screening of all compounds was performed against the reference and quality control strain Staphylococcus aureus ATCC 29213, three clinical isolates of methicillin-resistant S. aureus (MRSA). Interestingly, both antitumor and antimicrobial activity were shown to depend on the presence of substituents in the pyridoquinothiazine system. The compounds that showed the greatest antitumor activity were the derivatives **3d**, **3e** and **3j**, containing a chlorine atom, a fluorine atom or a methoxy group in the 9-position of the tetracyclic system, respectively. The highest antibacterial activity was revealed by compounds **3c** and **3e** bearing a bromine atom in the 10-position and a fluorine atom in the 9-position of the pyridoquinothiazine system. The previously described tetracyclic quinobenzothiazinium derivatives showed interesting anticancer properties, where the suggested mechanism of their antitumor activity is based on DNA intercalation. The newly synthesized pyridoquinothiazinium salts are structural analogs of quinobenzothiazines in which the benzene ring was replaced with a pyridine ring; therefore, the similar mechanism of action seems reasonable. As a matter of fact, some of the compounds show more potent anti-cell proliferation activity compared to the reference compound (cisplatinum). It appears that the flat structure of antiproliferative factor favors the intercalation into DNA (just as doxorubicin). The newly synthesized molecule **3a** with a completely flat tetracyclic motif showed noticeably weaker antiproliferative activity compared to isomer **3j** that is bent along the axis defined by the nitrogen and sulfur atoms of the thiazine ring. Consequently, it is not clear whether the new compounds inhibit cell proliferation only through DNA intercalation or other mechanisms of action exist as well. In this context, the actual molecular mechanism of action has to be clarified in future studies.

In silico computation of the intermolecular similarity was performed using the principal component analysis (PCA) and hierarchical clustering analysis (HCA) on the pool of structure/property-related descriptors calculated for novel tetracyclic diazaphenothiazine derivatives. The distance-oriented property evaluation for the congeneric series of compounds was correlated with experimentally measured anticancer activities and an empirical lipophilicity profile, respectively. A range of various software logP predictors for the estimation of the numerical lipophilic values was employed and subsequently cross-compared with the experimental parameters. Moreover, the newly synthesized adducts were subjected to the quantitative shape comparison (CoMSA) with the generation of an averaged pharmacophore pattern to illustrate the key 3D steric/electronic/lipophilic features. Finally, the quantitative sampling of similarity-related activity landscape (SALI) provided a subtle picture of favorable and disallowed structural modifications that are valid for determining the activity cliffs. Hopefully, the novel series of tetracyclic diazaphenothiazine derivatives with the functionalized pyridine subunit can be structurally modified to optimize anticancer activity and selectivity.

## Figures and Tables

**Table 1:** Structure of tetracyclic diazaphenothiazine derivatives **3a**–**j** and **4a** with experimentally determined log*P* and in vitro activities A549, SNB-19, T47D and NHDF.

		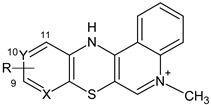						
Comp.	X	Y	R	log*P*	IC_50_ [μM]			
					A549 (std)	SNB-19 (std)	T47D (std)	NHDF (std)
**3a**	N	CH	H	3.53	70.3 ± 9.8	132.3 ± 11.6	46.2 ± 7.1	21.1 ± 1.5
**3b**	N	CH	9-Br	4.80	23.8 ± 1.7	4.1 ± 0.3	5.2 ± 0.4	0.8 ± 0.05
**3c**	N	C	10-Br	4.57	20.6 ± 1.2	3.4 ± 0.2	12.2 ± 1.7	11.0 ± 1.4
**3d**	N	CH	9-Cl	4.62	2.0 ± 0.3	1.3 ± 0.1	2.3 ± 0.3	7.7 ± 0.5
**3e**	N	CH	9-F	4.24	2.1 ± 0.35	0.4 ± 0.08	2.8 ± 0.35	1.1 ± 0.14
**3f**	N	CH	9-I	5.09	115.3 ± 10.7	62.5 ± 6.6	128.4 ± 18.2	97.4 ± 6.2
**3g**	N	CH	9-Cl, 11-CH_3_	5.66	20 ± 1.6	3.5 ± 0.2	26.7 ± 1.6	3.8 ± 0.3
**3h**	N	CH	9-F, 11-CH_3_	5.72	15.4 ± 1.0	125.5 ± 14.8	47.7 ± 2.8	28.5 ± 4.0
**3i**	N	CH	11-CH_3_	4.17	69.3 ± 5.3	22.1 ± 1.5	18.9 ± 1.4	0.4 ± 0.07
**3j**	N	CH	9-OCH_3_	4.20	1.7 ± 0.1	6.1 ± 0.4	8.1 ± 1.0	2.0 ± 0.3
**4a**	CH	N	H	2.93	30.1 ± 3.4	18.7 ± 1.2	36.8 ± 2.1	7.5 ± 0.2
cisplatinum	-	-	-	-	3.0 ± 0.2	16.7 ± 1.3	9.0 ± 0.7	29.9 ± 3.3

A549 (human lung carcinoma); SNB-19 (human glioblastoma); T47D (human breast cancer); NHDF (normal human dermal fibroblasts); std (standard deviation).

**Table 2:** Structure of **3a**–**j** and **4a**, in vitro anti-*Staphylococcus* activities MIC (μM) in comparison with ampicillin (AMP) standard.

Comp.	MIC (μM)			
	SA	MRSA 63718	MRSA SA 630	MRSA SA 3202
**3a**	212	212	106	212
**3b**	337	337	337	337
**3c**	42.1	42.1	21.1	42.1
**3d**	381	381	381	381
**3e**	50.2	25.1	12.5	25.1
**3f**	299	299	299	299
**3g**	>731	>731	>731	>731
**3h**	383	383	383	383
**3i**	203	203	101	203
**3j**	193	193	96	193
**4a**	424	424	424	424
**AMP**	5.72	45.8	45.8	45.8

SA = *Staphylococcus aureus* ATCC 29213; MRSA = clinical isolates of methicillin-resistant *S. aureus* 63718, SA 630, and SA 3202 (National Institute of Public Health, Prague, Czech Republic).

## Data Availability

Crystal data for structure (**3g**) and (**3j**) are available in the Cambridge Crystallographic Data Centre. CCDC deposition number 2095596 (**3g**). CCDC deposition number 2095597 (**3j**). https://ccdc.cam.ac.uk/.

## Supplementary Materials

The following are available online at https://www.mdpi.com/article/10.3390/ijms222312826/s1.
