# Supplementary material for: Towards Property Profiling: SYNTHESIS and SAR Probing of New Tetracyclic Diazaphenothiazine Analogues
Source: Int J Mol Sci. 2021 Nov 26;22(23):12826. doi: 10.3390/ijms222312826 (PMC8658022; doi:10.3390/ijms222312826)
Supplement: Supplementary file 1 [file ijms-22-12826-s001.zip › ijms-1473921-supplementary.pdf]

Supplementary Materials

Towards property profiling: Synthesis and SAR probing of new tetracyclic diazaphenothiazine analogues

Anna Empel [1], Andrzej Bak [2,*], Violetta Kozik [2], Malgorzata Latocha [3], Alois Cizek [4], Josef Jampilek [5,6], Kinga Suwinska [7], Aleksander Sochanik [8] and Andrzej Zieba [1,*]

[1] Department of Organic Chemistry, Faculty of Pharmaceutical Sciences in Sosnowiec, Medical University of Silesia in Katowice, Jagiellońska 4, 41-200 Sosnowiec, Poland; anna.empel00@gmail.com (A.E.); zieba@sum.edu.pl (A.Z.)
[2] University of Silesia, Institute of Chemistry, Szkolna 9, 40-007 Katowice, Poland; andrzej.bak@us.edu.pl (A.B.); violetta.kozik@us.edu.pl (V.K.)
[3] Department of Cell Biology, Faculty of Pharmaceutical Sciences in Sosnowiec, Medical University of Silesia in Katowice, Jedności 9, 41-200 Sosnowiec, Poland; mlatocha@sum.edu.pl (M.L.)
[4] Department of Infectious Diseases and Microbiology, Faculty of Veterinary Medicine, University of Veterinary Sciences Brno, Palackeho 1946/1, 61242 Brno, Czech Republic; cizeka@vfu.cz (A.C.)
[5] Department of Analytical Chemistry, Faculty of Natural Sciences, Comenius University, Ilkovicova 6, 842 15 Bratislava, Slovakia; josef.jampilek@gmail.com (J.J.)
[6] Department of Chemical Biology, Faculty of Science, Palacky University Olomouc, Slechtitelu 27, 783 71 Olomouc, Czech Republic
[7] Faculty of Mathematics and Natural Sciences, Cardinal Stefan Wyszyński University, K. Woycickiego 1/3, 01-938 Warszawa, Poland; k.suwinska@uksw.edu.pl (K.S.)
[8] Center for Translational Research and Molecular Biology of Cancer, Maria Skłodowska-Curie National Research Institute of Oncology, Wybrzeże AK 15, 44-101 Gliwice, Poland; aleksander.sochanik@io.gliwice.pl (A.S.)
* Correspondence: zieba@sum.edu.pl (A.Z.); andrzej.bak@us.edu.pl (A.B.)

Figure S1. ^{1}H NMR Spectrum in CD_3OD of compound **3a**.

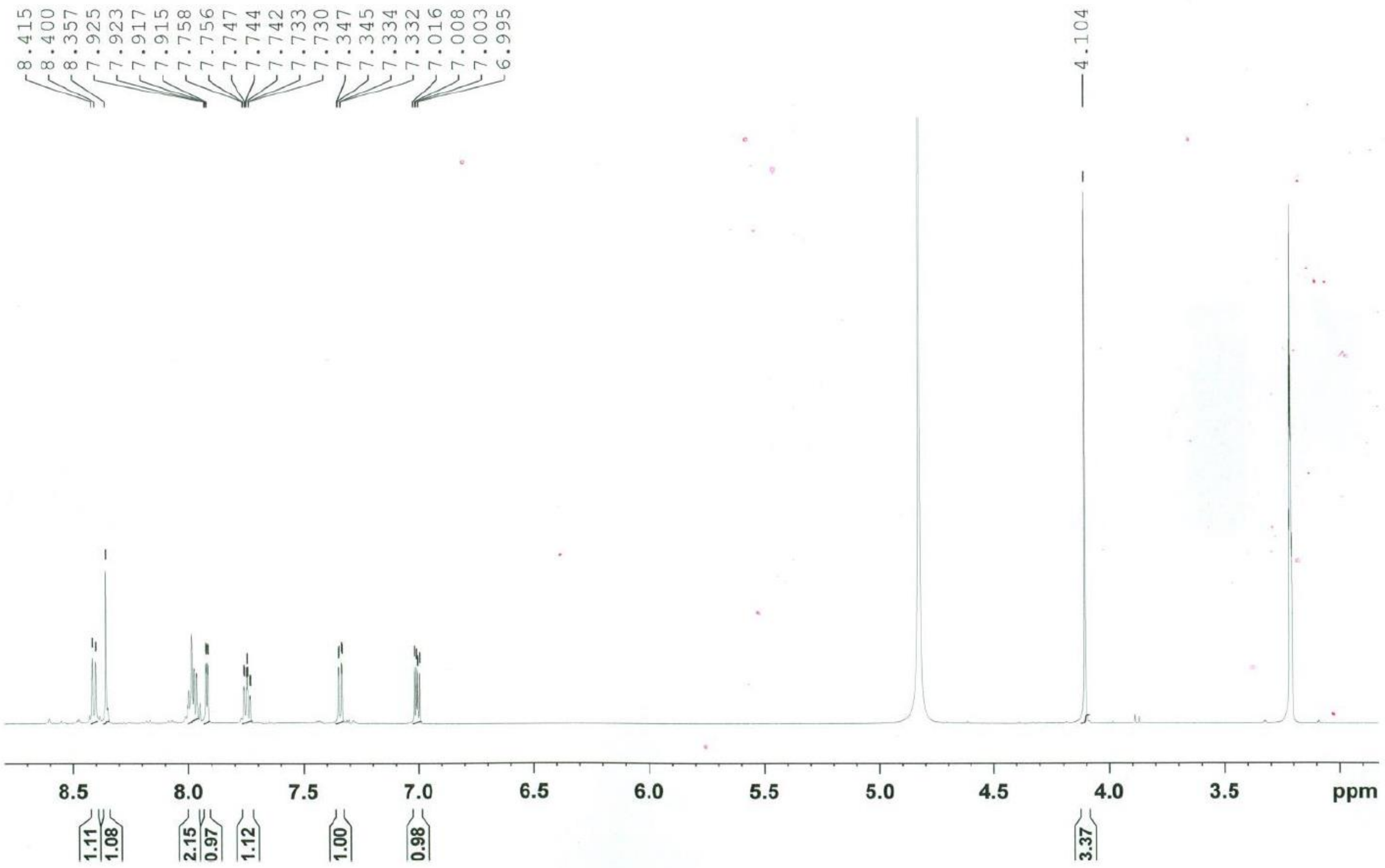

Figure S2. ^{13}C NMR Spectrum in CD3OD of compound **3a.**

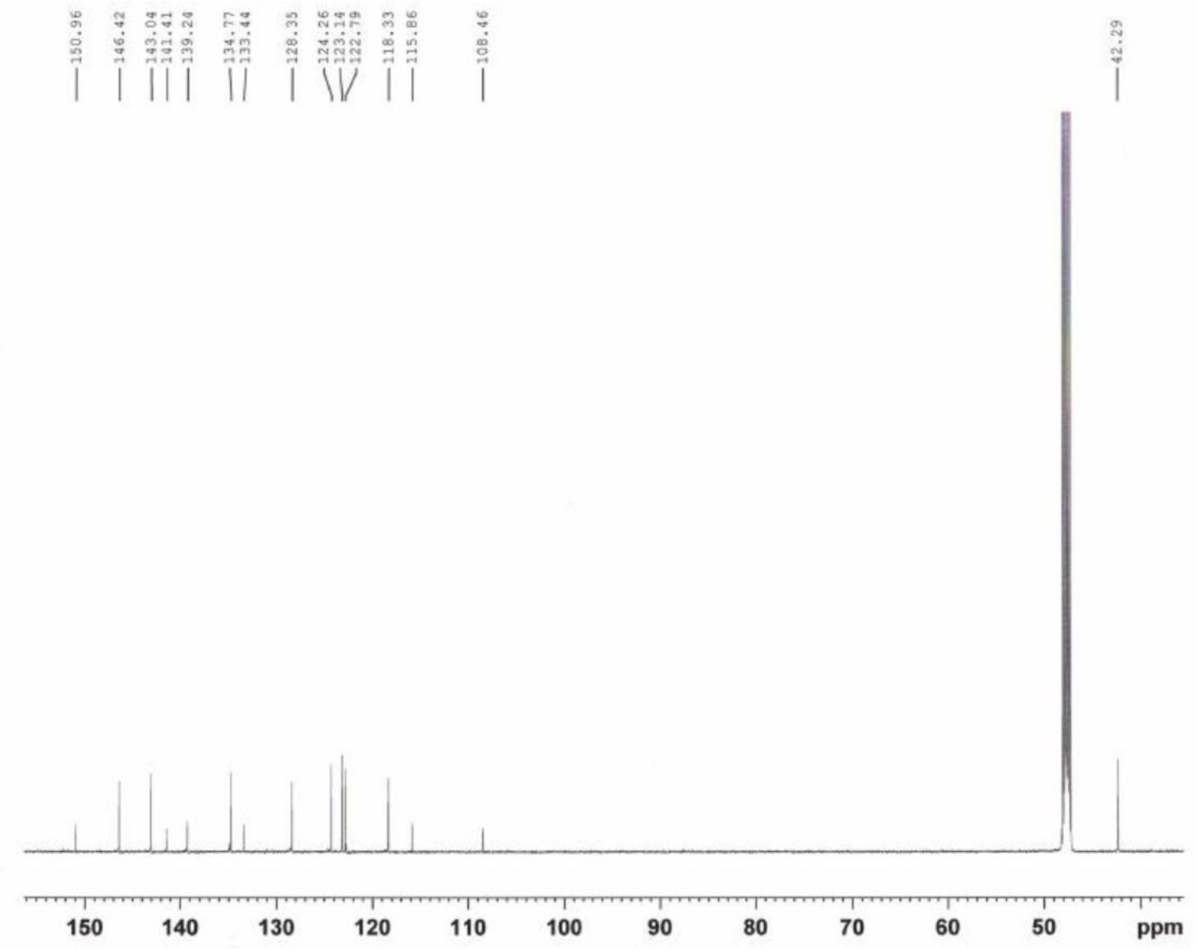

Figure S3. ^{1}H NMR Spectrum in CD_3OD of compound **3b.**

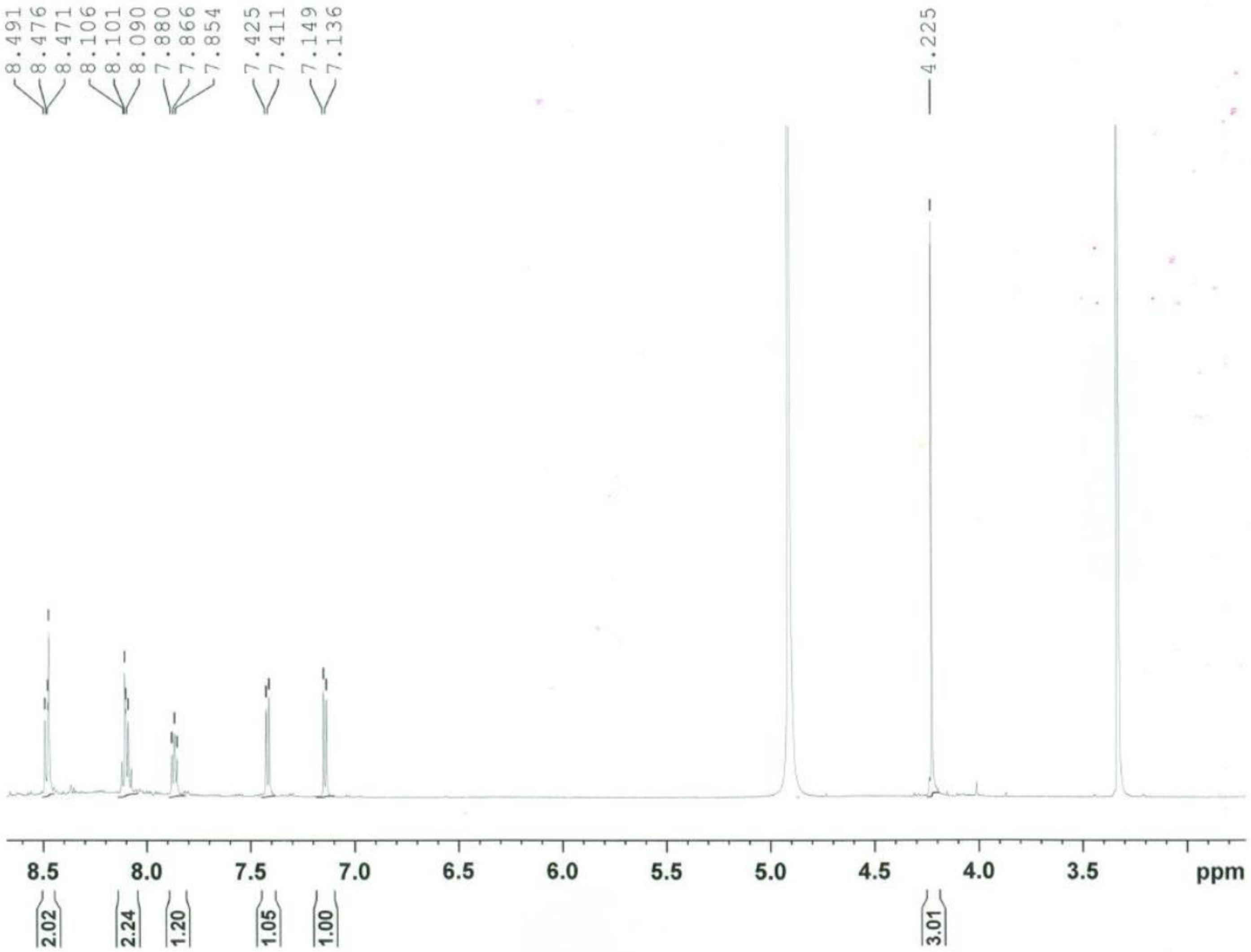

Figure S4. ^{13}C NMR Spectrum in CD3OD of compound **3b.**

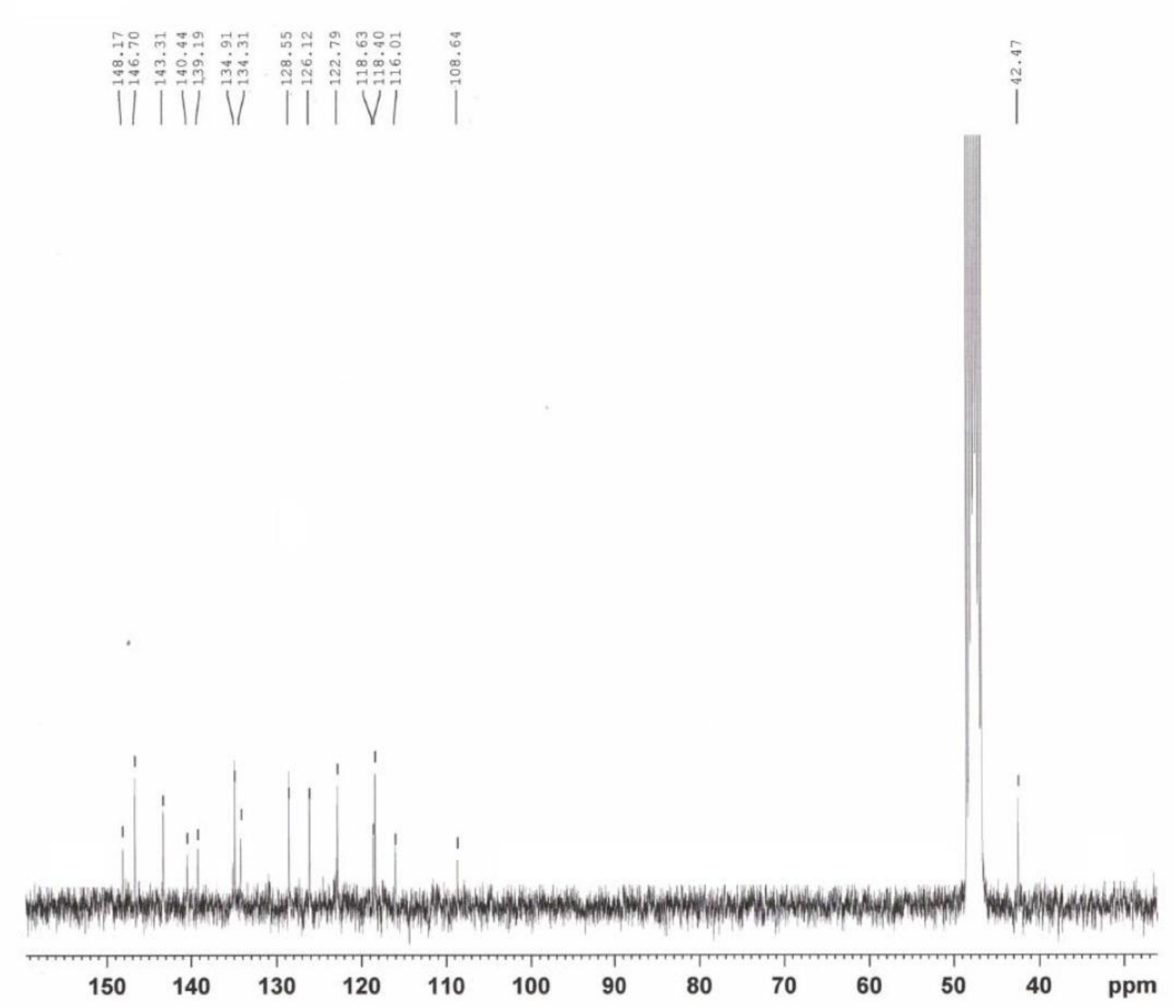

Figure S5. ^1H NMR Spectrum in CD_3OD of compound **3c.**

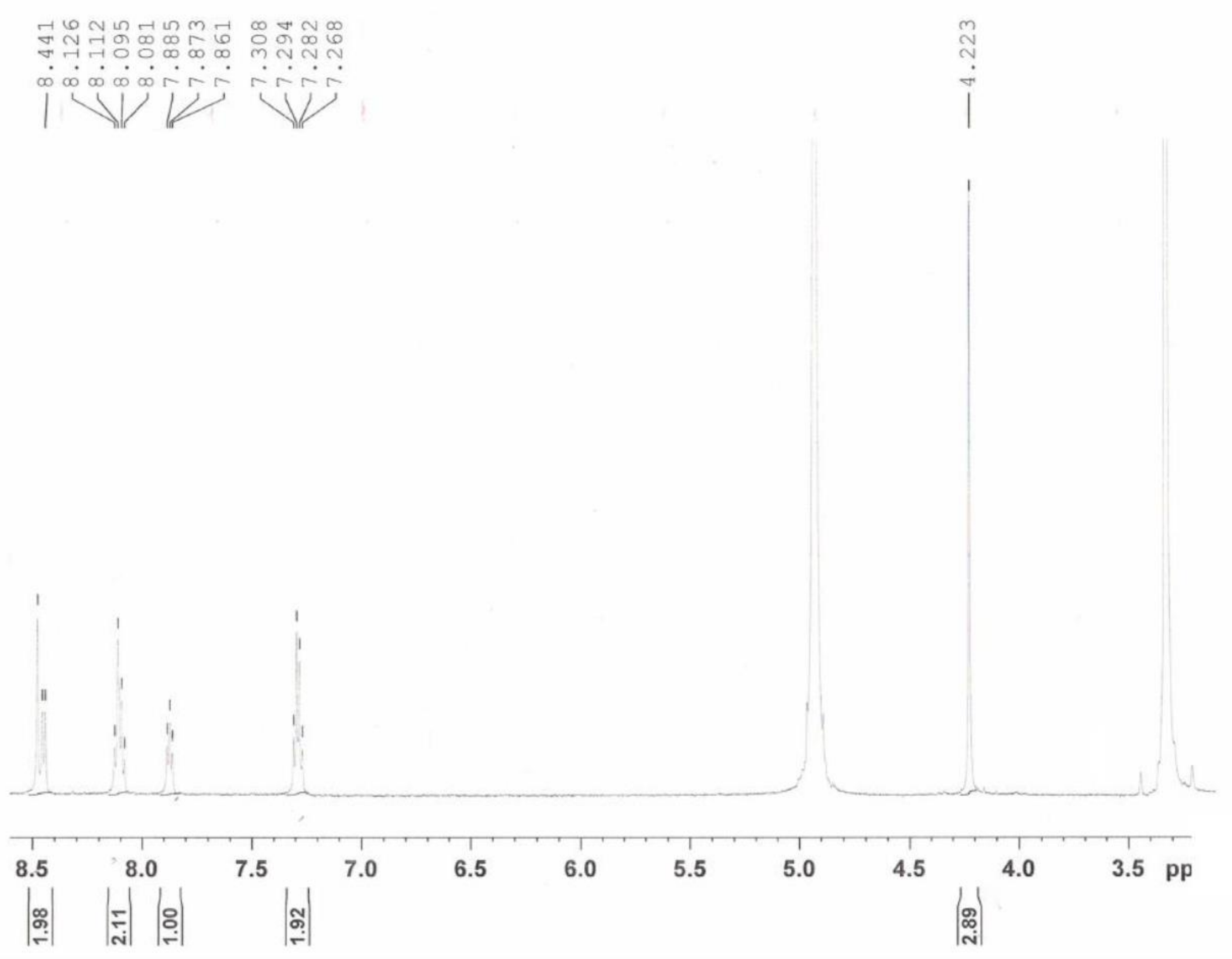

Figure S6. ^{13}C NMR Spectrum in CD3OD of compound **3c.**

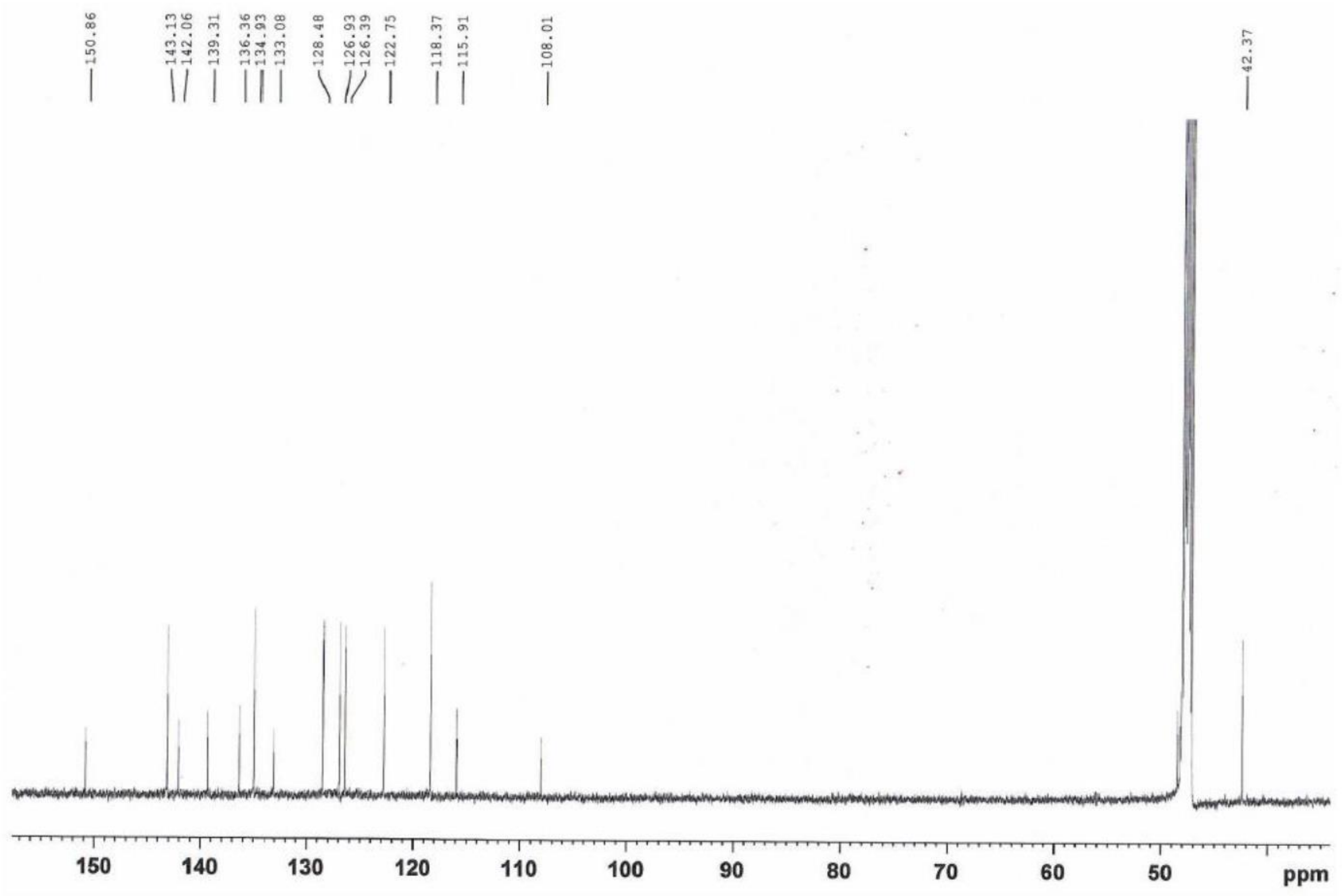

Figure S7. ^{1}H NMR Spectrum in CD_3OD of compound **3d.**

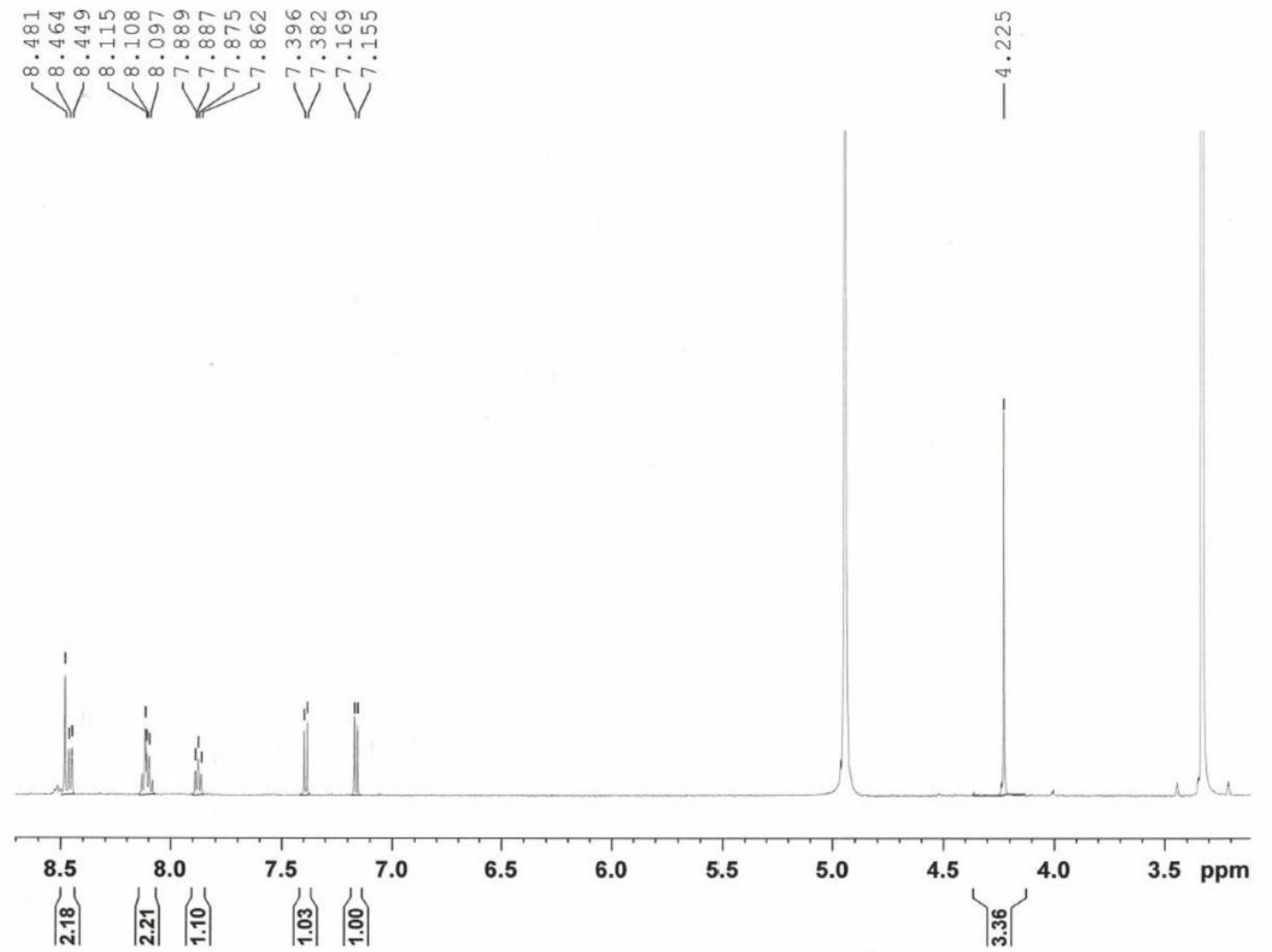

Figure S8. ^{13}C NMR Spectrum in CD3OD of compound **3d.**

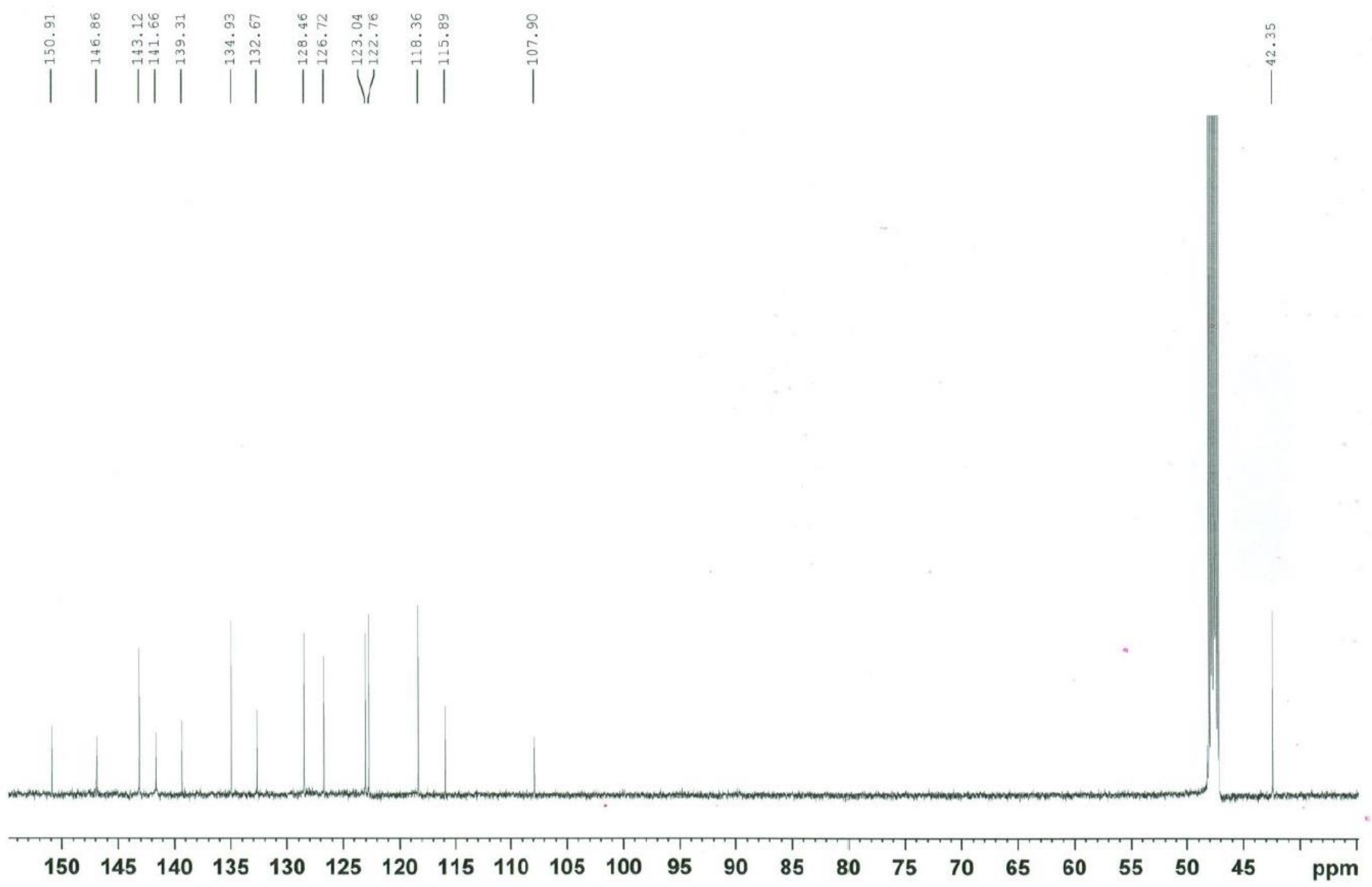

Figure S9. ^{1}H NMR Spectrum in CD_3OD of compound **3e.**

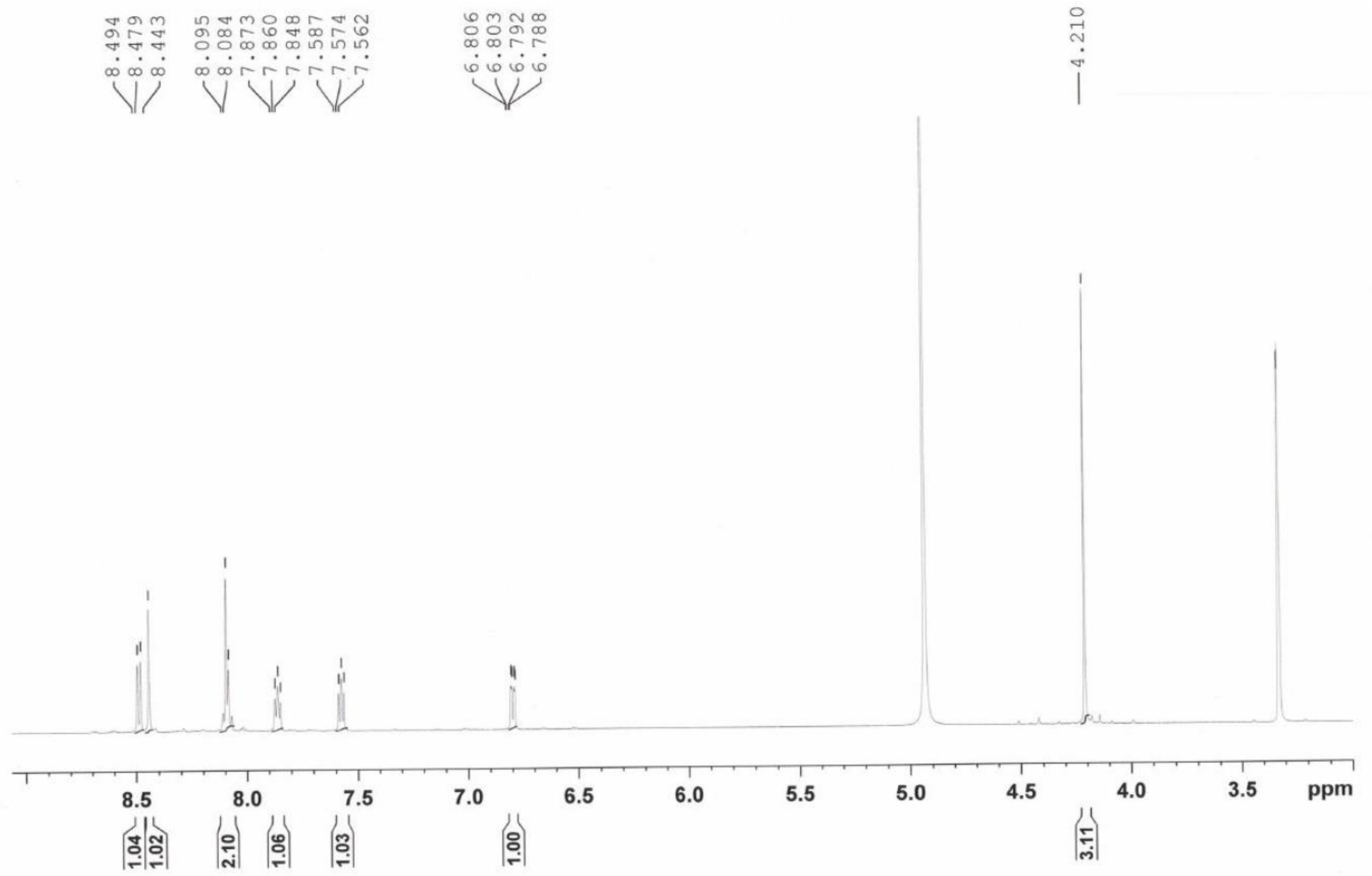

Figure S10. ^{13}C NMR Spectrum in CD3OD of compound **3e.**

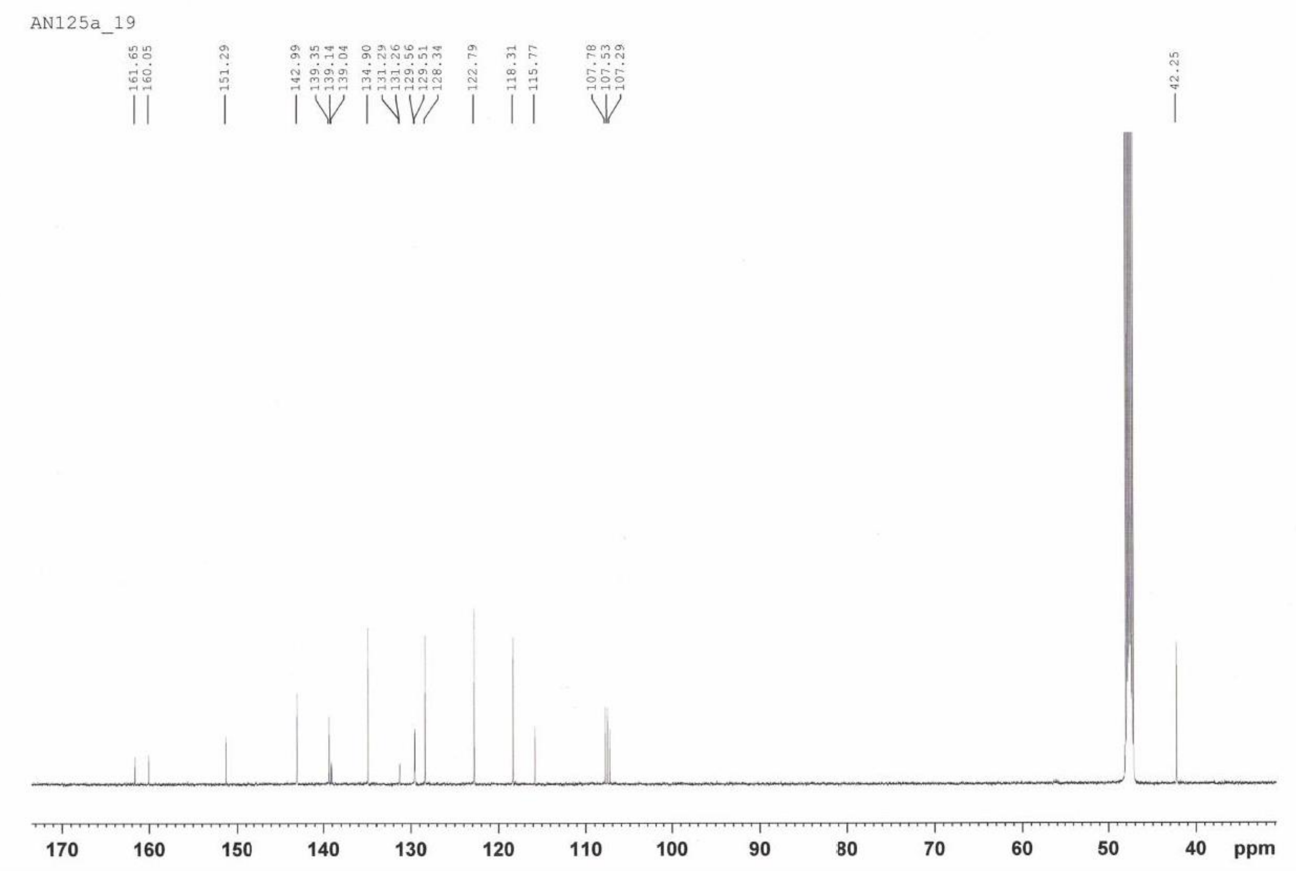

Figure S11. ^{1}H NMR Spectrum in CD_3OD of compound **3f.**

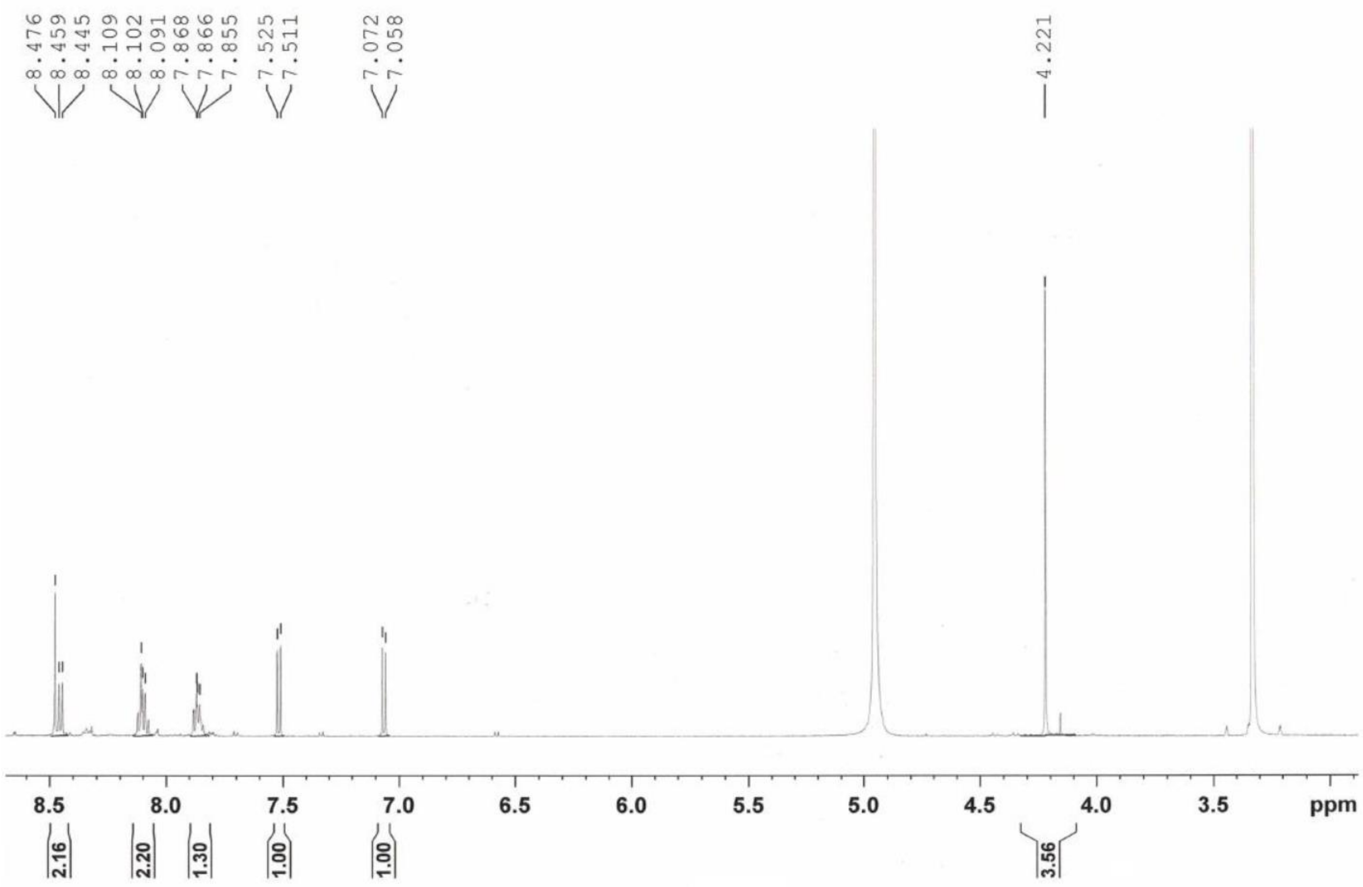

Figure S12. ^{13}C NMR Spectrum in CD3OD of compound **3f.**

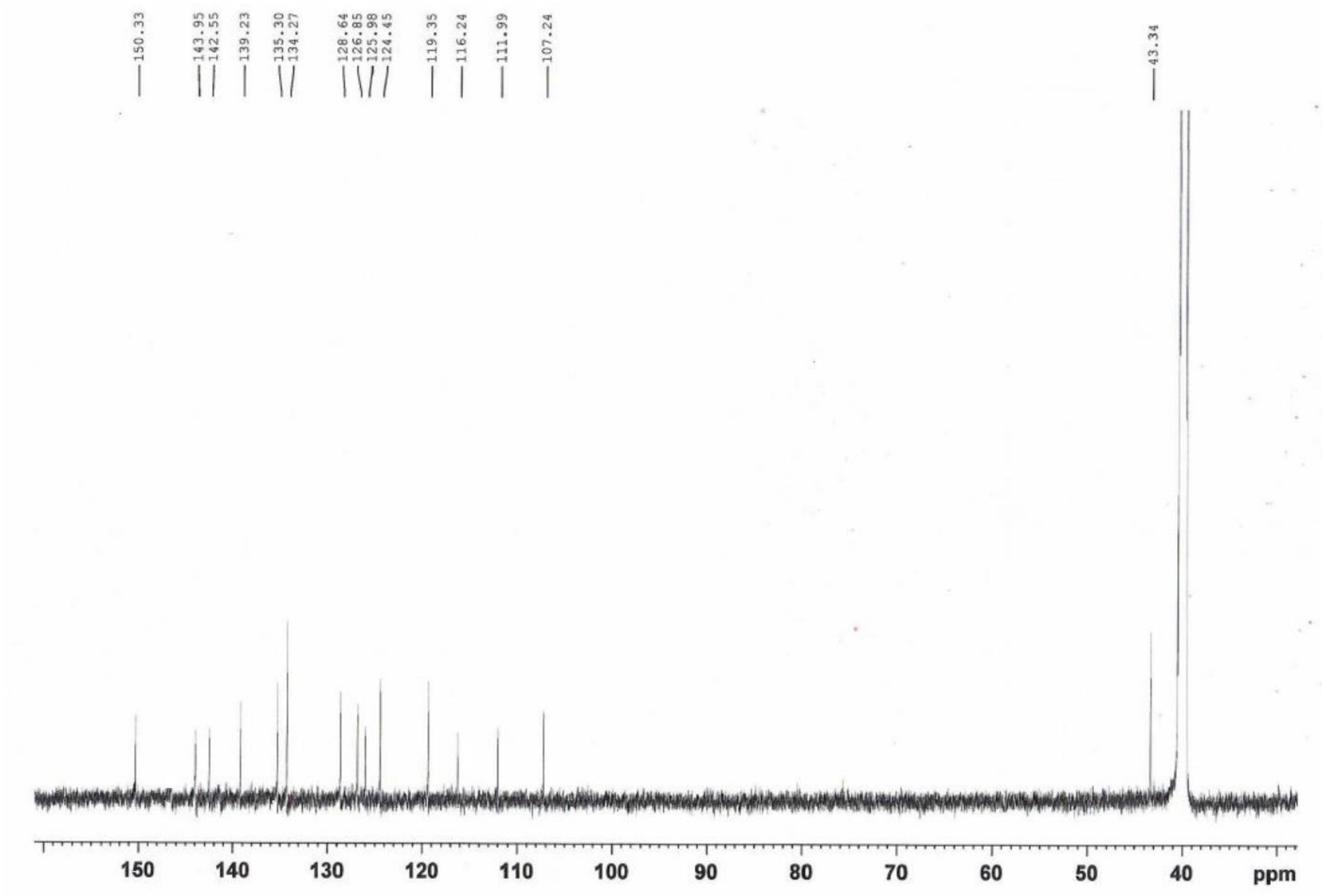

Figure S13. ^{1}H NMR Spectrum in CD_3OD of compound **3g.**

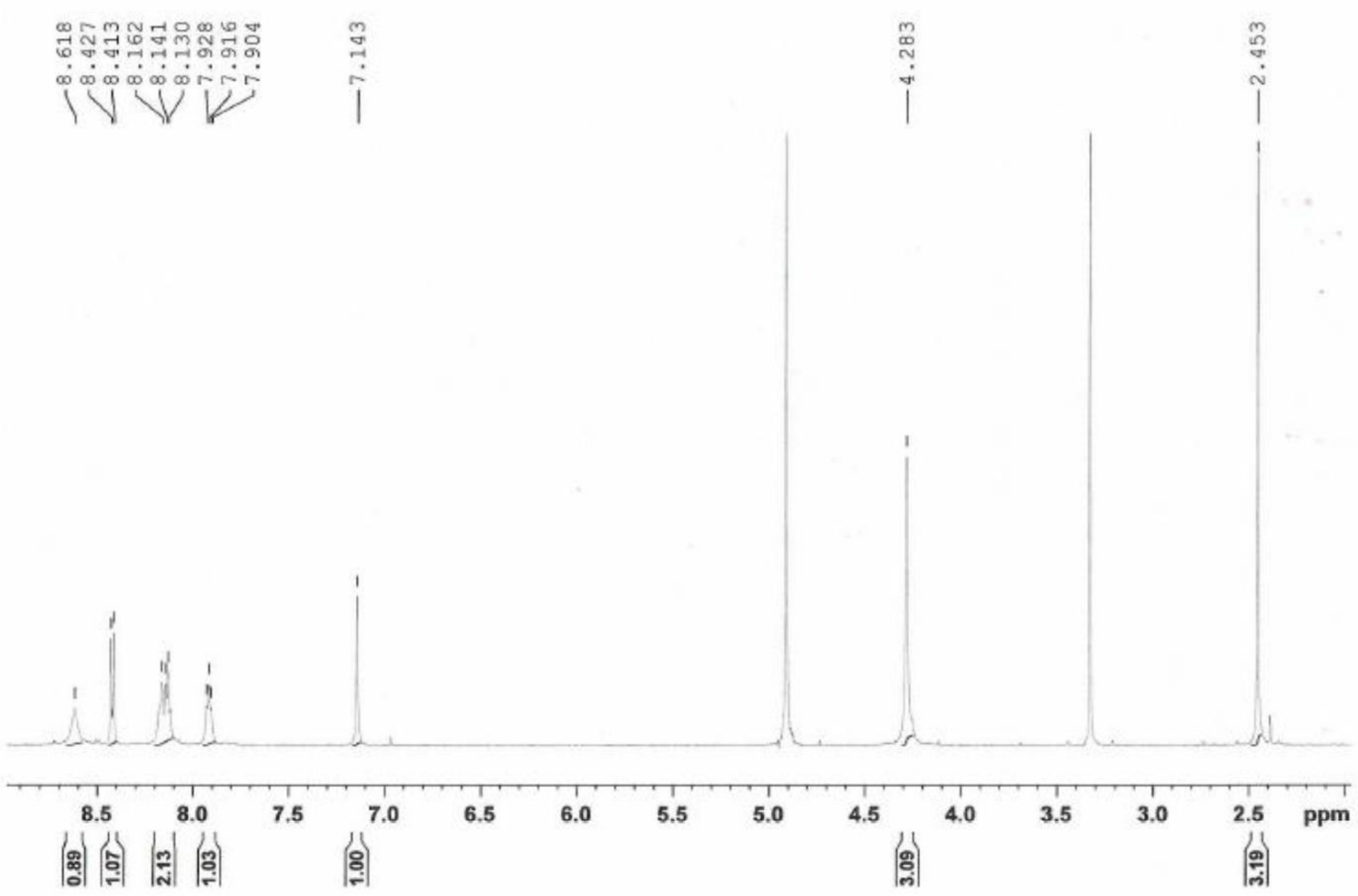

Figure S14. ^{13}C NMR Spectrum in CD3OD of compound **3g.**

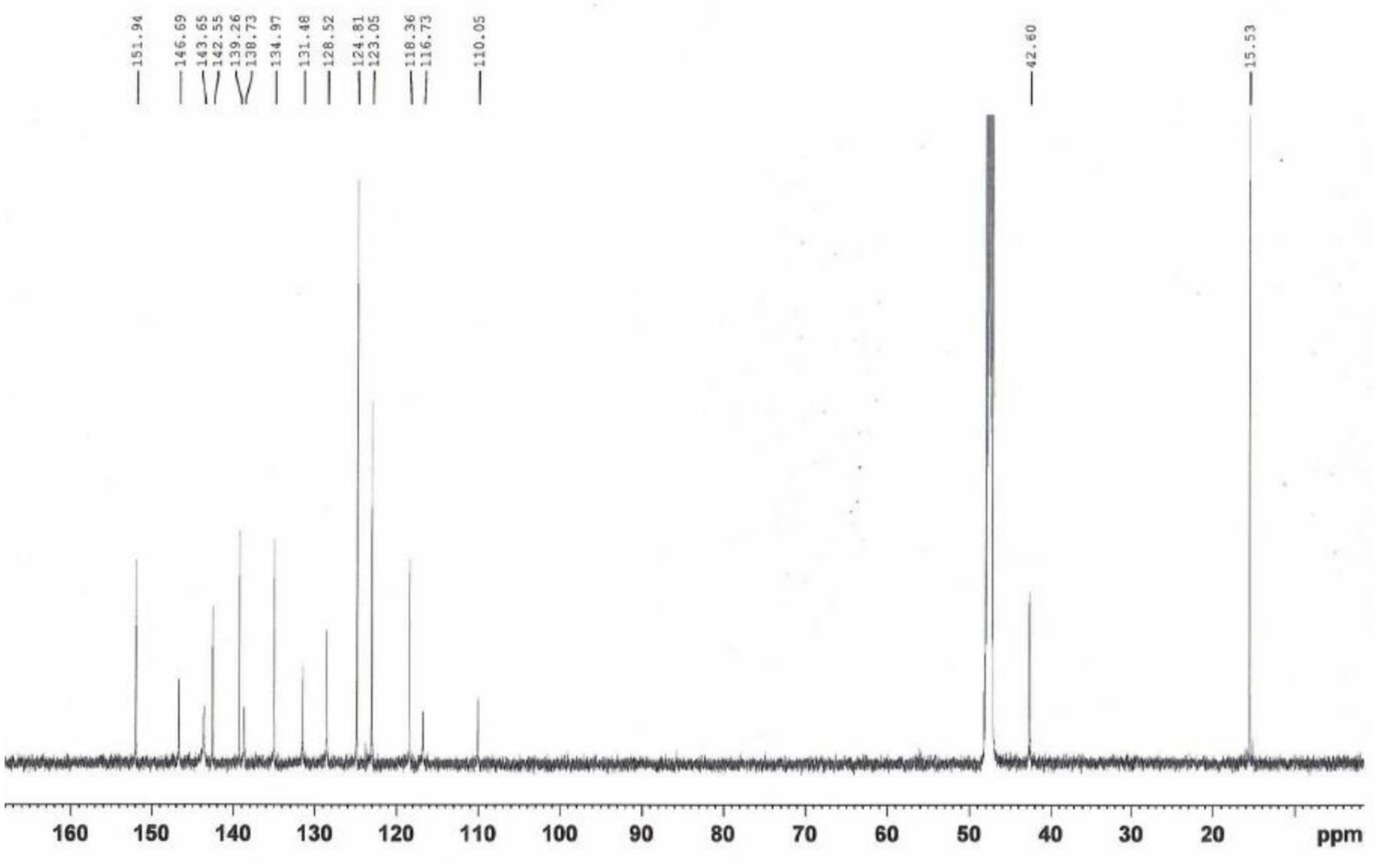

Figure S15. ^{1}H NMR Spectrum in CD_3OD of compound **3h.**

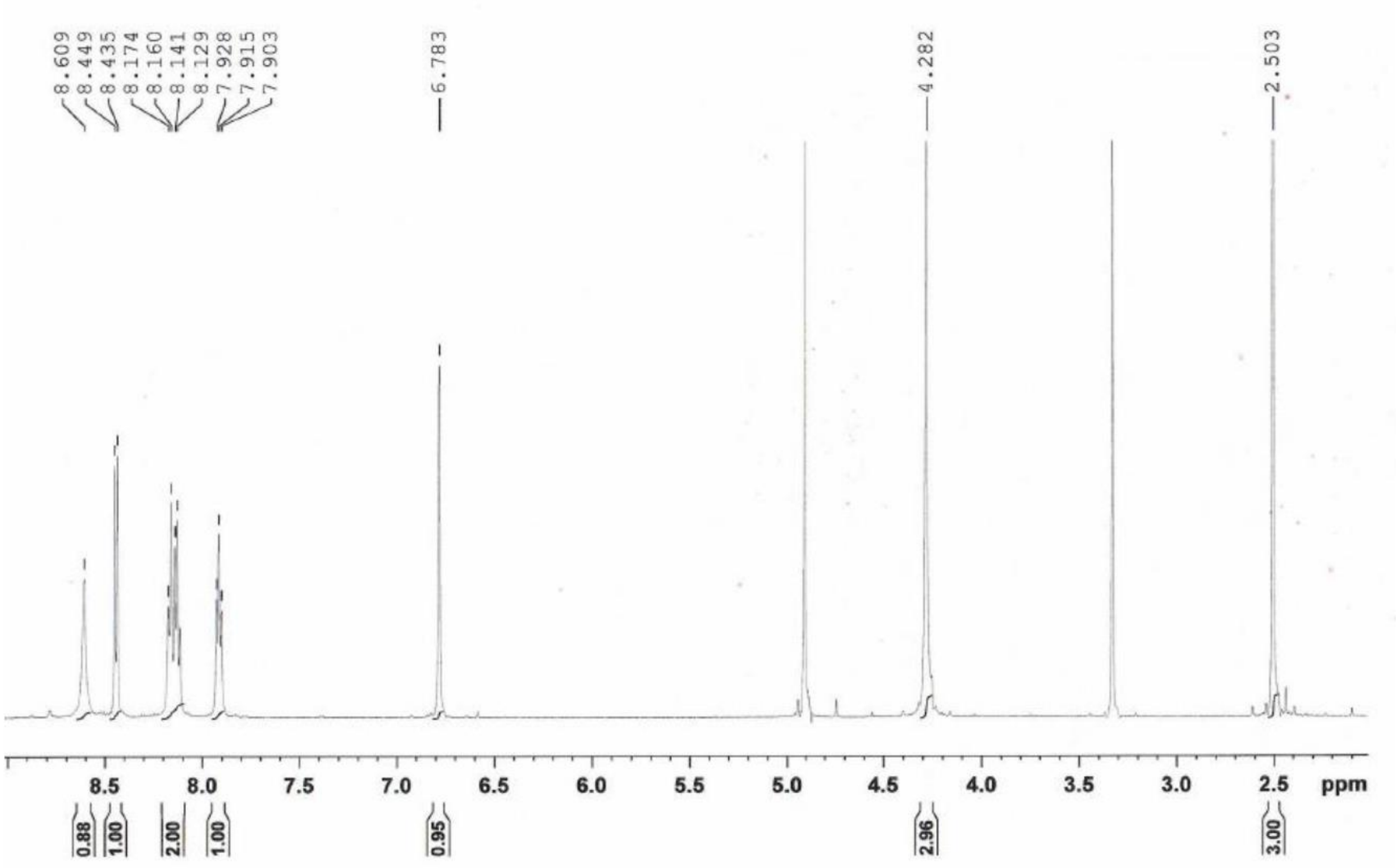

Figure S16. ^{13}C NMR Spectrum in CD3OD of compound **3h.**

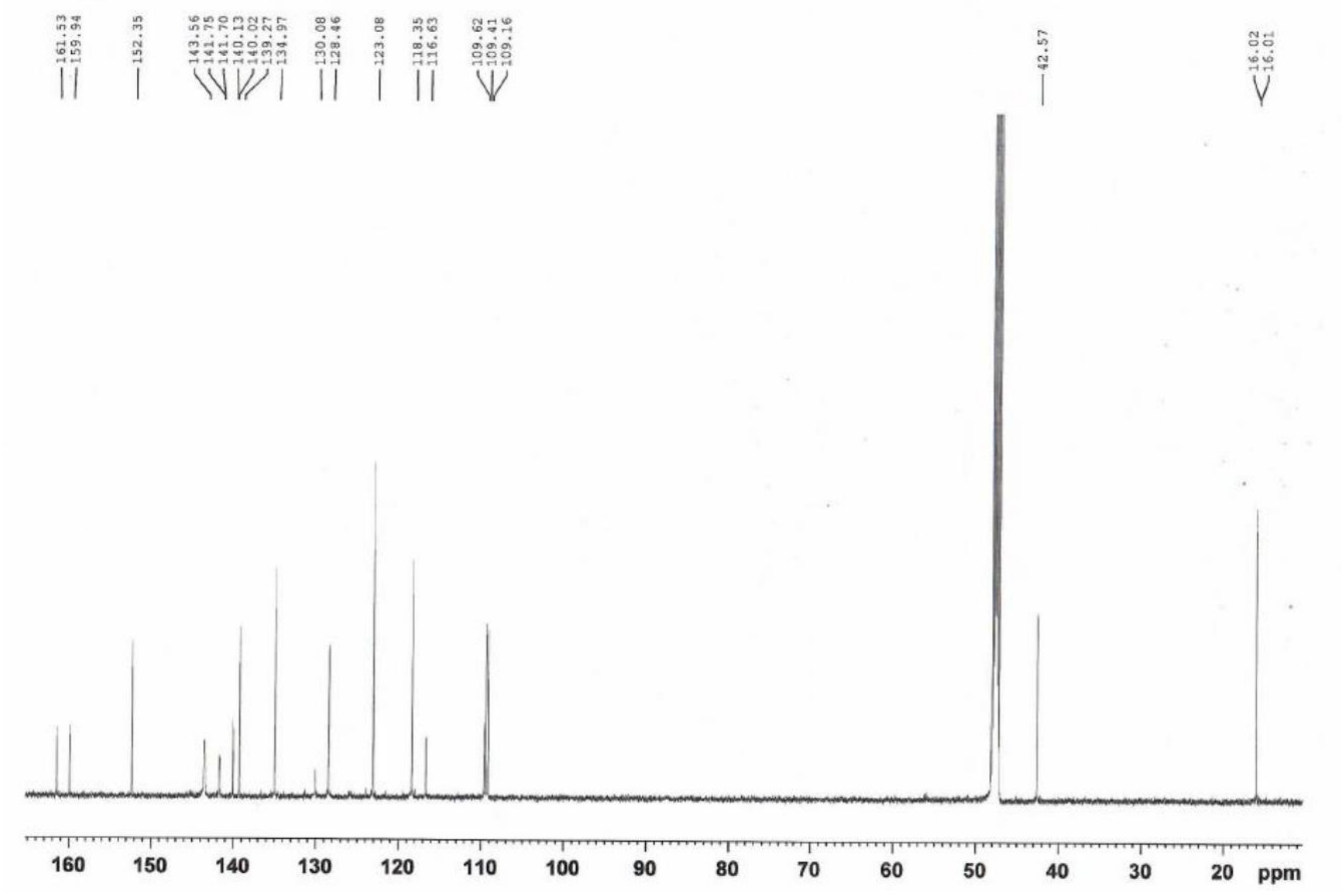

Figure S17. ^{1}H NMR Spectrum in CD_3OD of compound **3i.**

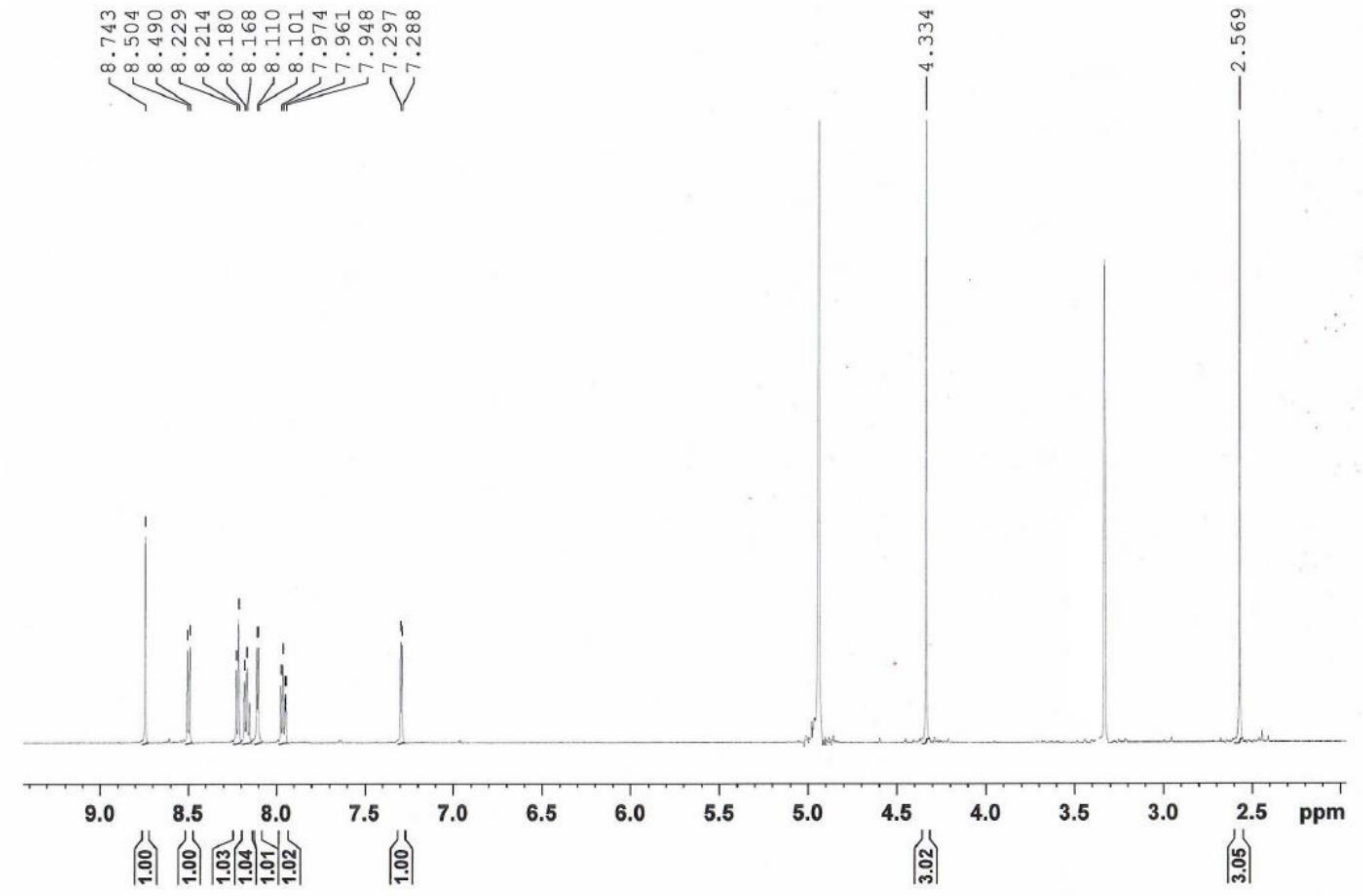

Figure S18. ^{13}C NMR Spectrum in CD3OD of compound **3i.**

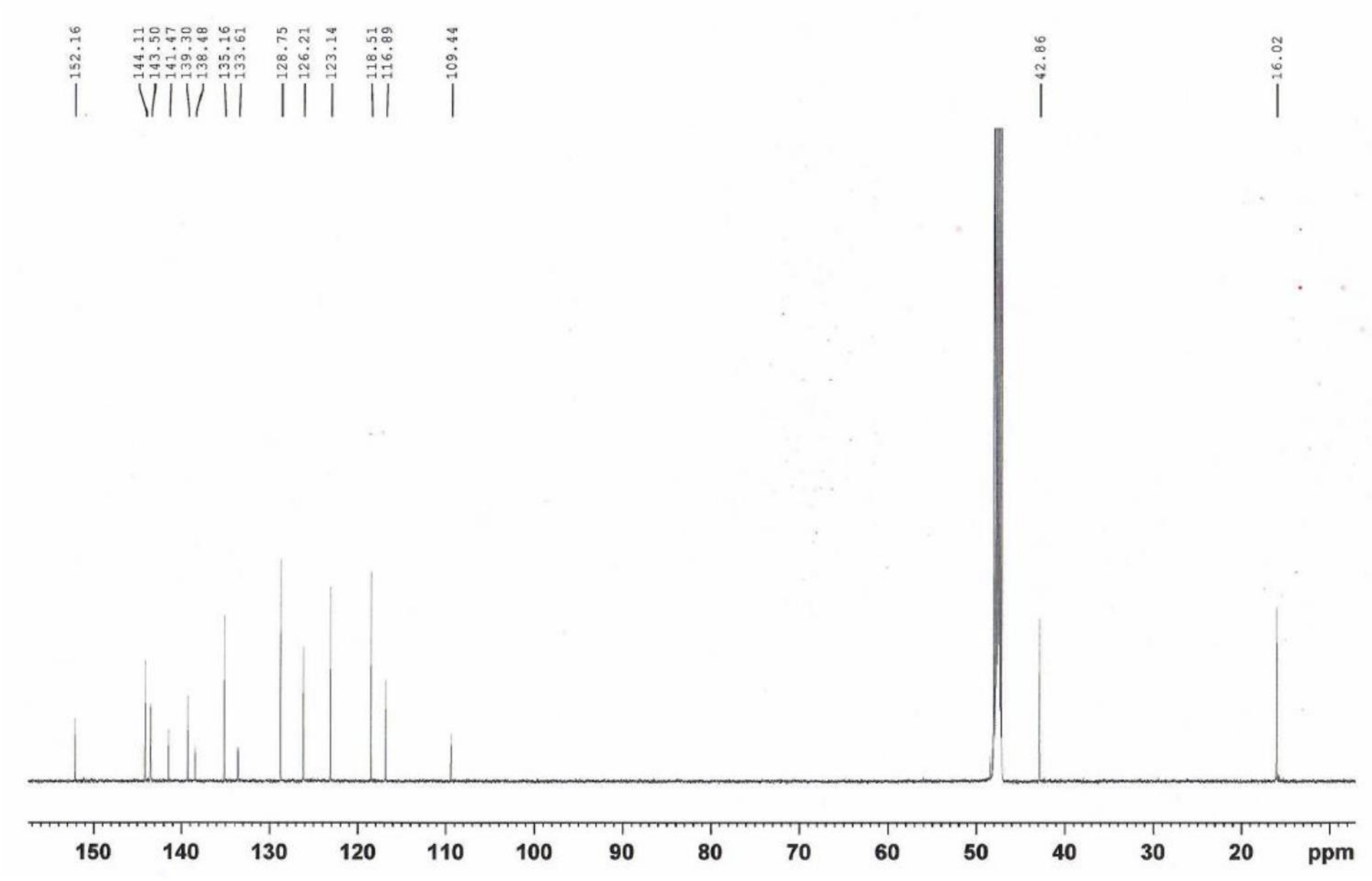

Figure S19. ^{1}H NMR Spectrum in CD_3OD of compound **3j.**

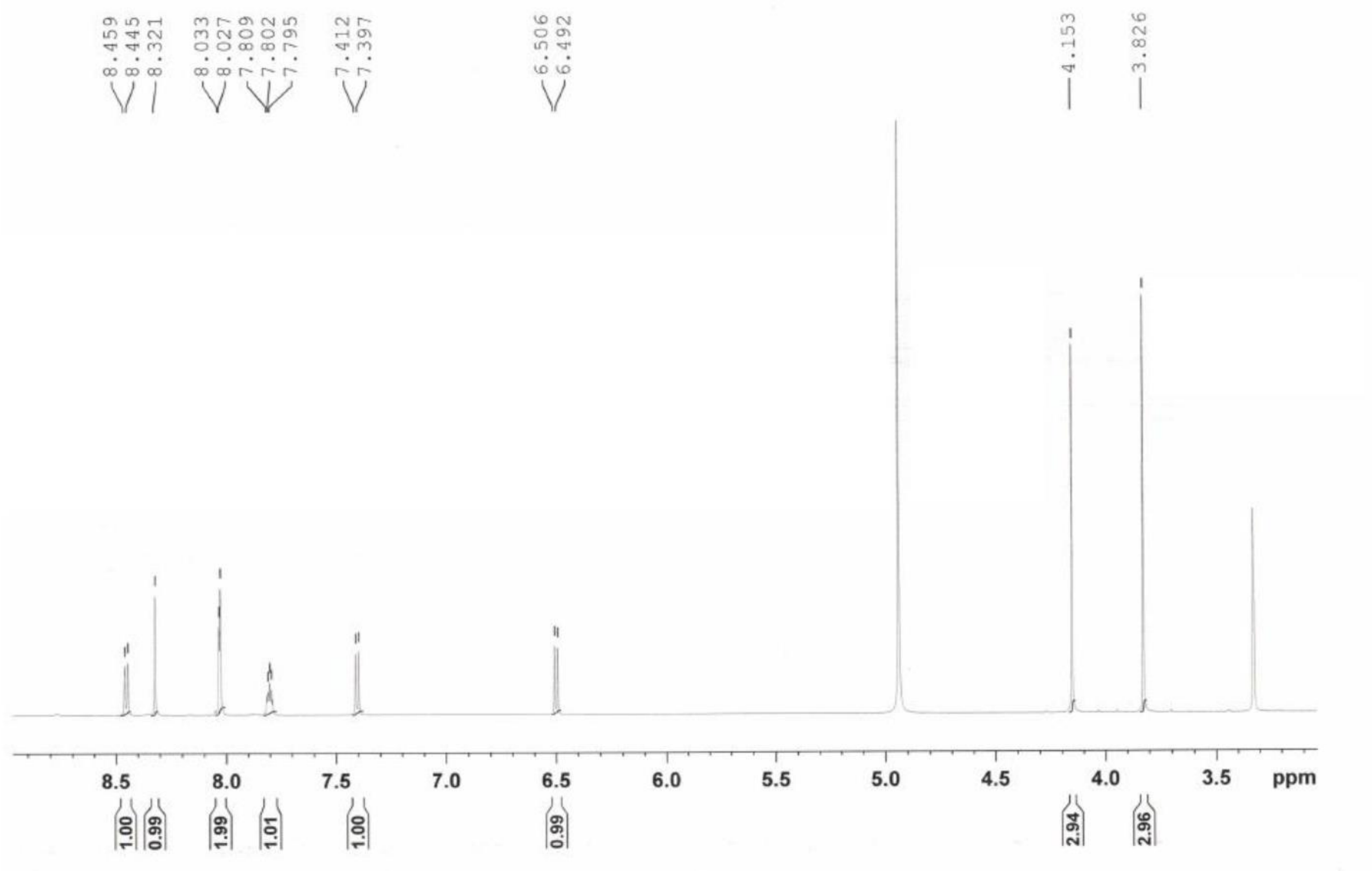

Figure S20. ^{13}C NMR Spectrum in CD3OD of compound **3j.**

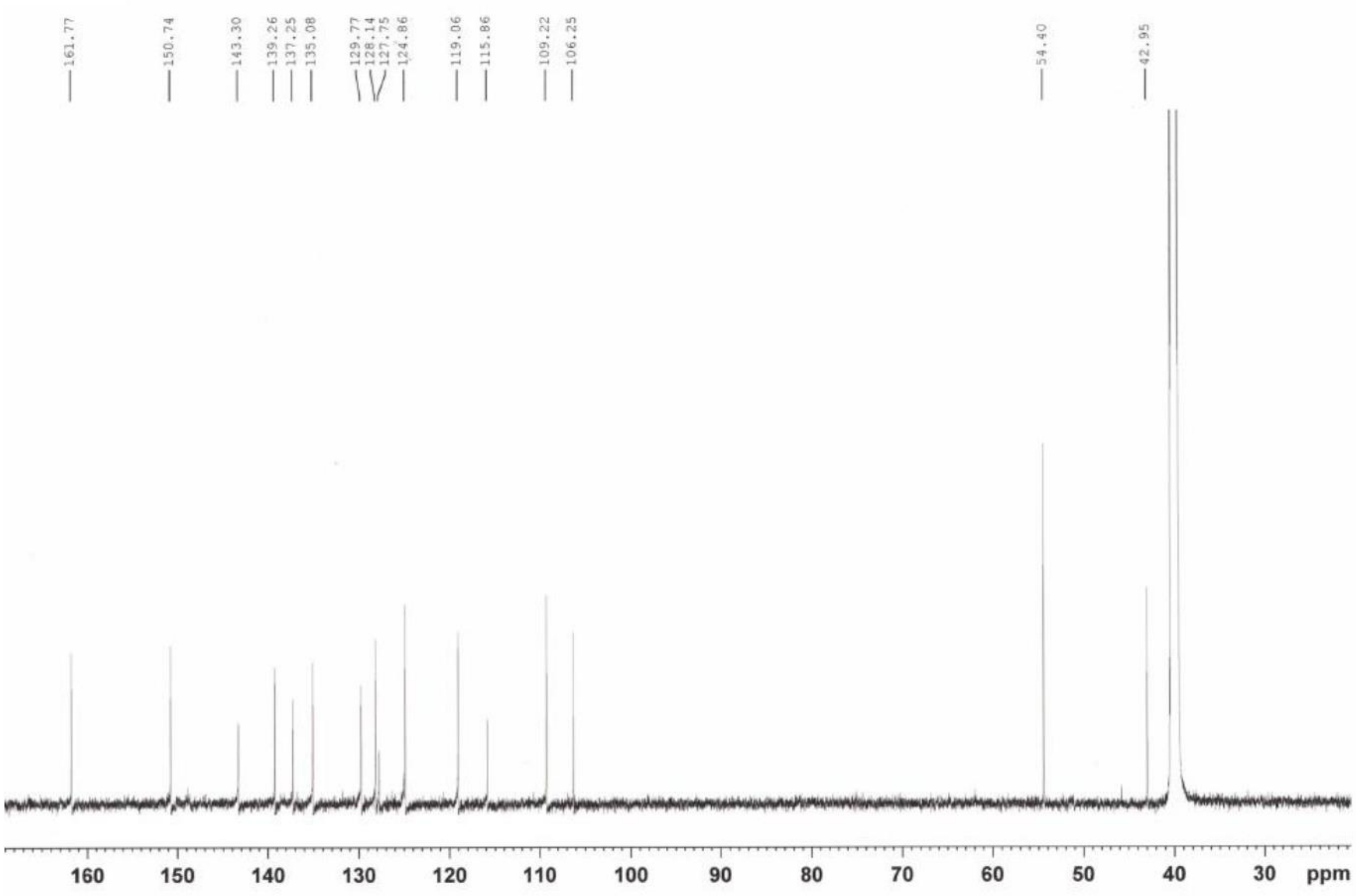

Figure S21. ^{1}H NMR Spectrum in CD_3OD of compound **4a.**

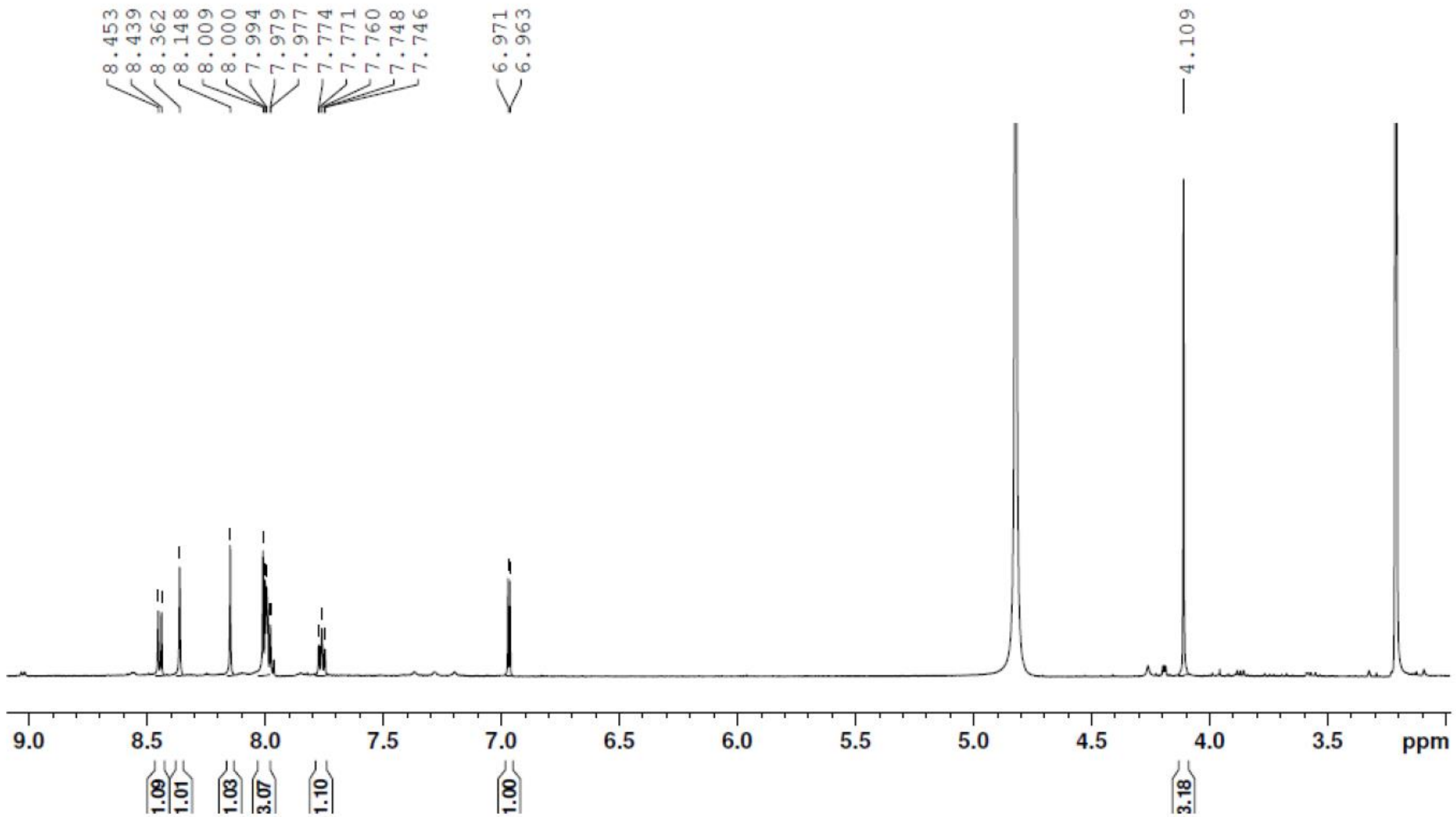

Figure S22. ^{13}C NMR Spectrum in CD3OD of compound **4a.**

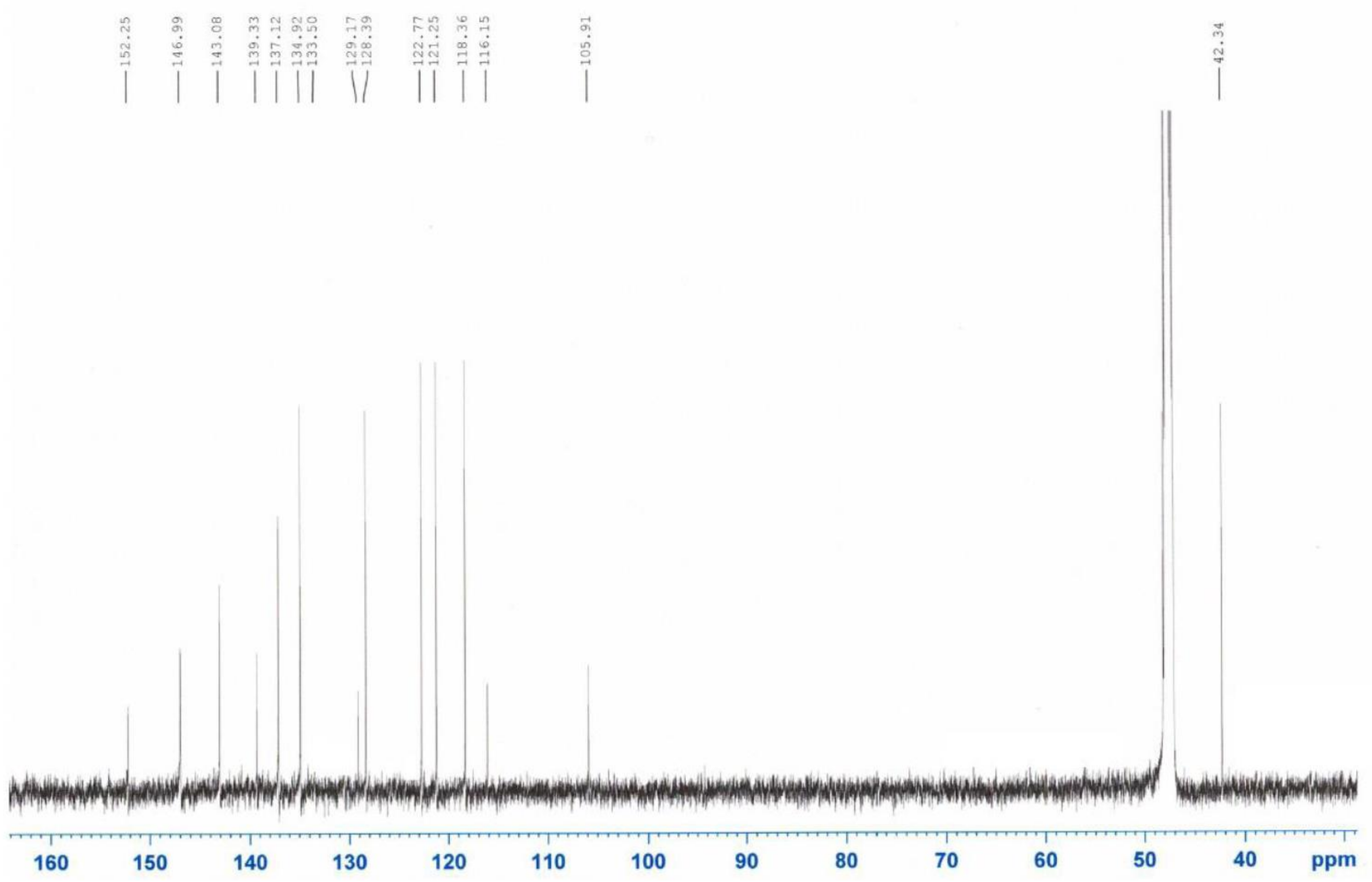

Lonza

Lonza Walkersville, Inc.
www.lonza.com
scientific.support@lonza.com
Scientific Support: 800-521-0390
Document # CC-31-6 11/10
Walkersville, MD 21793-0127 USA
© 2010 Lonza Walkersville, Inc.

Clonetics™ Dermal Fibroblast Cell Systems

NHDF

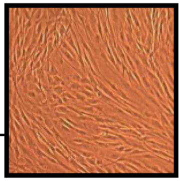

Introduction

Clonetics™ Dermal Fibroblast Cell Systems contain Normal Human Dermal Fibroblasts (NHDF) and optimized media for their growth. Each System can quickly generate NHDF cultures for the study of disorders of human fibroblasts such as fibrosis, scleroderma, fibrosarcoma, xeroderma pigmentosum and histiocytoma. Clonetics™ Dermal Fibroblast Cell Systems are convenient and easy to use, allowing the researcher to focus on results. Cryopreserved NHDF are shipped as frozen primaries. Proliferating NHDF are shipped as secondary cultures.

Clonetics™ Cells, Medium and Reagents are quality tested together and guaranteed to give optimum performance as a complete Cell System.

Cell System Components (Need to be purchased separately)

- One Dermal Fibroblast Cell Product (Cryopreserved or Proliferating)
- One Fibroblast Cell Medium BulletKit™ - 500 ml

  Clonetics™ FGM™-2 BulletKit™ (CC-3132) contains one 500 ml bottle of Fibroblast Cell Basal Medium and the following growth supplements: hFGF-B, 0.5 ml; Insulin, 0.5 ml; FBS, 10 ml; GA-1000, 0.5 ml.
- One ReagentPack™ (CC-5034) Containing:

Trypsin/EDTA	100 ml
Trypsin Neutralizing Solution	100 ml
HEPES Buffered Saline Solution	100 ml

Characterization of Cells

Routine characterization of NHDF includes morphological observation throughout serial passages.

Performance

Recommended seeding density for subculture	3,500 cells/cm^2
Typical time from subculture to confluent monolayer	6 - 9 days
Additional population doublings guaranteed using Clonetics™ System	15

Quality Control

All cells are performance assayed and test negative for HIV-1, mycoplasma, Hepatitis-B, Hepatitis-C, bacteria, yeast and fungi. Cell viability, morphology and proliferative capacity are measured after recovery from cryopreservation. Clonetics™ Media are formulated for optimal growth of specific types of normal human cells. Certificates of Analysis (COA) for each cell strain are shipped with each order. COA for all other products are available upon request.

Ordering Information

Cryopreserved Cells

CC-2511	NHDF-Ad	≥500,000 cells
CC-2509	NHDF-Neo	≥500,000 cells

Proliferating Cells – Flasks and Multiwell Plates

NHDF-Ad

CC-2611	T-25 Flask
CC-0252	T-75 Flask
CC-0160	96-well Plate

NHDF-Neo

CC-2609	T-25 Flask
CC-0210	T-75 Flask
CC-0116	96-well Plate

Other proliferating formats are available. Contact Scientific Support or refer to the Lonza website for details.

CC-3132	FGM™-2 BulletKit™, FBM™ plus SingleQuots™ of Growth Supplements	500 ml
CC-3131	FBM™, Fibroblast Basal Medium	500 ml
CC-4126	FGM™-2 SingleQuots™, Formulates FBM™ to FGM™-2	
CC-5034	ReagentPack™	
	Trypsin/EDTA Solution	100 ml
	Trypsin Neutralizing Solution	100 ml
	HEPES Buffered Saline Solution	100 ml

When placing an order or for technical service, please refer to the product numbers and descriptions listed above. For a complete listing of all Clonetics™ Products, refer to the Lonza website or the current Lonza catalog. To obtain a catalog, additional information or technical service you may contact Lonza by web, e-mail, telephone, fax or mail.

Product Warranty

CULTURES HAVE A FINITE LIFESPAN IN VITRO. Lonza guarantees the performance of its cells only if Clonetics™ Media and Reagents are used exclusively, and the recommend protocols are followed. The performance of cells is not guaranteed

Lonza

THESE PRODUCTS ARE FOR RESEARCH USE ONLY. Not approved for human or veterinary use, for application to humans or animals, or for use in clinical or in vitro procedures.

WARNING: CLONETICS™ AND POIETICS™ PRODUCTS CONTAIN HUMAN SOURCE MATERIAL, TREAT AS POTENTIALLY INFECTIOUS. Each donor is tested and found non-reactive by an FDA approved method for the presence of HIV-I, Hepatitis B Virus and Hepatitis C Virus. Where donor testing is not possible, cell products are tested for the presence of viral nucleic acid from HIV, Hepatitis B Virus, and Hepatitis C Virus. Testing can not offer complete assurance that HIV-1, Hepatitis B Virus, and Hepatitis C Virus are absent. All human sourced products should be handled at the Biological Safety Level 2 to minimize exposure of potentially infectious products, as recommended in the CDC-NIH Manual, Biosafety in Microbiological and Biomedical Laboratories, 5th Edition. If you require further information, please contact your site Safety Officer or Scientific Support.
